# Dynamic and adaptive cancer stem cell population admixture in colorectal neoplasia

**DOI:** 10.1016/j.stem.2022.07.008

**Published:** 2022-08-04

**Authors:** Ester Gil Vazquez, Nadia Nasreddin, Gabriel N. Valbuena, Eoghan J. Mulholland, Hayley L. Belnoue-Davis, Holly R. Eggington, Ryan O. Schenck, Valérie M. Wouters, Pratyaksha Wirapati, Kathryn Gilroy, Tamsin R.M. Lannagan, Dustin J. Flanagan, Arafath K. Najumudeen, Sulochana Omwenga, Amy M.B. McCorry, Alistair Easton, Viktor H. Koelzer, James E. East, Dion Morton, Livio Trusolino, Timothy Maughan, Andrew D. Campbell, Maurice B. Loughrey, Philip D. Dunne, Petros Tsantoulis, David J. Huels, Sabine Tejpar, Owen J. Sansom, Simon J. Leedham

**Affiliations:** 1 Wellcome Centre Human Genetics, Roosevelt Drive, University of Oxford, Oxford, UK; 2 Laboratory for Experimental Oncology and Radiobiology, Center for Experimental and Molecular Medicine, Amsterdam University Medical Centers, Meibergdreef 9, 1105 Amsterdam, the Netherlands; 3 Oncode Institute, Meibergdreef 9, 1105 Amsterdam, the Netherlands; 4 Swiss Institute for Bioinformatics, University of Lausanne, Lausanne, Switzerland; 5 Cancer Research UK Beatson Institute, Glasgow, UK; 6 Centre for Cancer Research and Cell Biology, Queen’s University Belfast, Belfast, UK; 7 Department of Oncology, Old Road Campus Research Building, Roosevelt Drive, University of Oxford, Oxford, UK; 8 Department of Pathology and Molecular Pathology, University and University Hospital Zürich, Rämistrasse 100, 8006 Zürich, Switzerland; 9 Translational Gastroenterology Unit, John Radcliffe Hospital, University of Oxford, and Oxford NIHR Biomedical Research Centre, Oxford, UK; 10 Academic Department of Surgery, University of Birmingham, Birmingham, UK; 11 Candiolo Cancer Institute FPO IRCCS, 10060 Candiolo, Torino, Italy; 12 University of Geneva and Department of Oncology, Hôpitaux Universitaires de Genève, Geneva, Switzerland; 13 Molecular Digestive Oncology Unit, KU Leuven, Leuven, Belgium; 14 Institute of Cancer Sciences, University of Glasgow, Garscube Estate, Glasgow, UK

**Keywords:** stem cells, cell plasticity, colorectal neoplasia, molecular phenotyping, intestinal stem cells, intestinal polyps, colorectal cancer

## Abstract

Intestinal homeostasis is underpinned by *LGR5+*ve crypt-base columnar stem cells (CBCs), but following injury, dedifferentiation results in the emergence of *LGR5*−ve regenerative stem cell populations (RSCs), characterized by fetal transcriptional profiles. Neoplasia hijacks regenerative signaling, so we assessed the distribution of CBCs and RSCs in mouse and human intestinal tumors. Using combined molecular-morphological analysis, we demonstrate variable expression of stem cell markers across a range of lesions. The degree of CBC-RSC admixture was associated with both epithelial mutation and microenvironmental signaling disruption and could be mapped across disease molecular subtypes. The CBC-RSC equilibrium was adaptive, with a dynamic response to acute selective pressure, and adaptability was associated with chemoresistance. We propose a fitness landscape model where individual tumors have equilibrated stem cell population distributions along a CBC-RSC phenotypic axis. Cellular plasticity is represented by position shift along this axis and is influenced by cell-intrinsic, extrinsic, and therapeutic selective pressures.

## Highlights

• Tumors have *Lgr5*+ve crypt base columnar (CBC) and *Lgr5*−ve regenerative stem cells (RSCs)
• Stem cell admixture can be measured from transcriptional data using the stem cell index
• Stem phenotypes vary by genotype and subtype, shifting with cell signaling disruption
• Phenotype shifts from selective pressures associate with poor response to chemotherapy

## Graphical abstract

**Figure:**
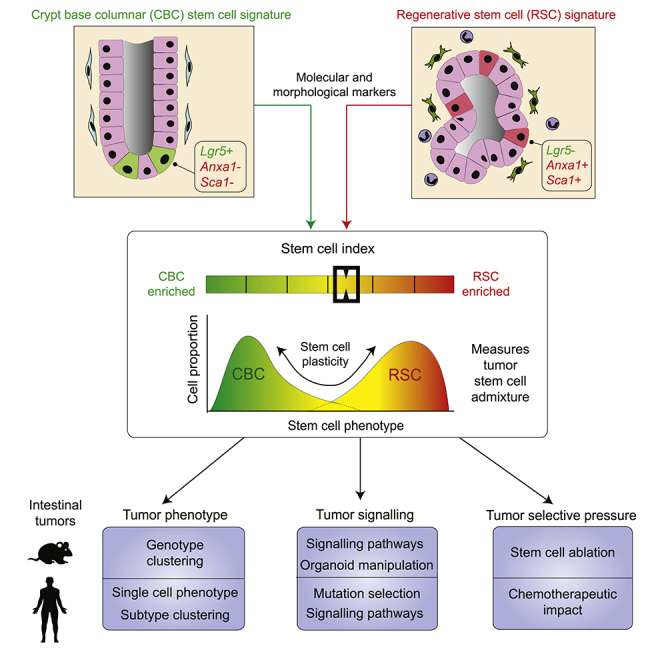

## Teaser

Gil Vazquez et al. demonstrate that intestinal tumors contain variable populations of stem cells whose admixture can be assessed using a transcriptional molecular tool—the stem cell index. Tumor stem cell molecular phenotype associated with lesion subtype and cell-intrinsic and extrinsic signaling disruption and shifted responsively to tumor selective pressures.

## Introduction

The intestine is an exemplar of an adult stem cell-supported tissue system. The identification of selective expression of *Leucine-rich repeat-containing G-protein coupled receptor 5* (*Lgr5*) in the crypt-base columnar cells (CBCs) of both human and murine crypts enabled lineage tracing and the demonstration of the first *bona fide* intestinal stem cell marker (Barker et al., 2007). Although *Lgr5+*ve CBCs demonstrably underpin steady-state intestinal homeostasis, their immediate contribution in supporting epithelial regeneration is less clear. *Lgr5* expression declines to undetectable levels following colitis induction in murine models (Davidson et al., 2012) and recovers at day 5 post injury, suggesting that (an) alternative stem cell population(s) support the early response to epithelial damage. Recent work has shown dedifferentiation and adaptive reprogramming of multiple cell types within residual epithelium (de Sousa and de Sauvage, 2019), with reversion to a primitive molecular phenotype and induction of fetal intestinal gene expression through activation of epithelial Yap signaling (Nusse et al., 2018; Yui et al., 2018). Individual genes within this fetal transcriptional program, such as *Ly6a* (*Sca1*), *Anxa1* (Nusse et al., 2018; Yui et al., 2018), and *Clu* (Ayyaz et al., 2019), have been used to identify regenerative cells with stem cell properties (collectively termed regenerative stem cells, or RSCs, from here on out).

Cancer is often described as the “wound that never heals” through co-option and corruption of physiological cell signaling. Neoplasia is characterized by the activity of stem cells, from the impact of initiating (epi)mutation through to the emergence of therapy-resistant clones and metastatic seeding (Fumagalli et al., 2020). In mouse models, genetic inactivation of the key colorectal cancer (CRC) driver gene *Adenomatous Polyposis Coli* (*Apc*) in *Lgr5*+ve cells precipitated rapid tumor induction, confirming CBCs as a cell-of-origin in intestinal tumorigenesis (Barker et al., 2009). However, murine studies subsequently showed that induction of inflammation and disruption of homeostatic morphogen gradients could result in neoplasia originating from *Lgr5*−ve cells, outside of the crypt base (Schwitalla et al., 2013; Davis et al., 2015). Furthermore, selective and effective killing of *Lgr5* cells had no impact on primary tumor growth (de Sousa e Melo et al., 2017) and the migratory cells that seed and colonize distant organs were frequently *Lgr5*−ve at dissemination (Fumagalli et al., 2020). In humans, recent integrated analysis of single-cell data demonstrated that serrated polyps arise from differentiated cells through a gastric metaplastic process (Chen et al., 2021), and a proportion of established colorectal tumors have minimal expression of *LGR5* (Merlos-Suarez et al., 2011; Morral et al., 2020; Shimokawa et al., 2017). Elegant recent work has shown that subpopulations both of *Lgr5*+ve and −ve tumor cells have elevated rDNA transcription and protein synthesis characteristic of functional stem cell activity (Morral et al., 2020) and that lineage conversion between cell types can be driven by the combination of key CRC driver genes and microenvironmental extracellular signaling (Han et al., 2020). Together these data indicate (1) the presence of alternative/additional (*Lgr5*−ve) stem cell populations in neoplastic lesions, and (2) that induced cell plasticity allows primary tumors to adapt to the loss of individual cancer stem cell populations.

Natural selection acts upon phenotype, so the capacity to easily measure a definable and pathologically relevant cancer molecular phenotype that can temporally track cancer cell fate is central to the concept of assessing tumor evolutionary trajectory. Stem cell plasticity underpins intestinal regeneration, and it is evident that *Lgr5* cannot be used as a sole marker for putative cancer stem cell populations in established lesions. Here we have undertaken molecular and morphological analysis to assess the stem cell molecular phenotype across a range of mouse neoplasia models, derived organoids, and human lesions and examine the factors that influence phenotypic plasticity. We propose a conceptual phenotypic fitness landscape model to contextualize the relationship between neoplastic stem cell activity (fitness) and cellular phenotype. *Lgr5*+ve CBCs and *Lgr5*−ve RSCs represent distinct but interlinked fitness peaks along a stem cell phenotypic axis. In individual untreated tumors, there is a distribution of stem cell phenotypes along this axis that reaches an equilibrium point, determined by combination of selected epithelial mutation and microenvironmental signaling. Phenotypic plasticity can be represented by a shifting in the phenotype distribution of the stem cell population, is regulated by emergent adaptive signaling pathways, and is required for tumor adaptation to therapeutic selective pressures.

## Results

### Application of molecular signatures and morphological markers to identify intestinal stem cell populations

**Figure 1:**
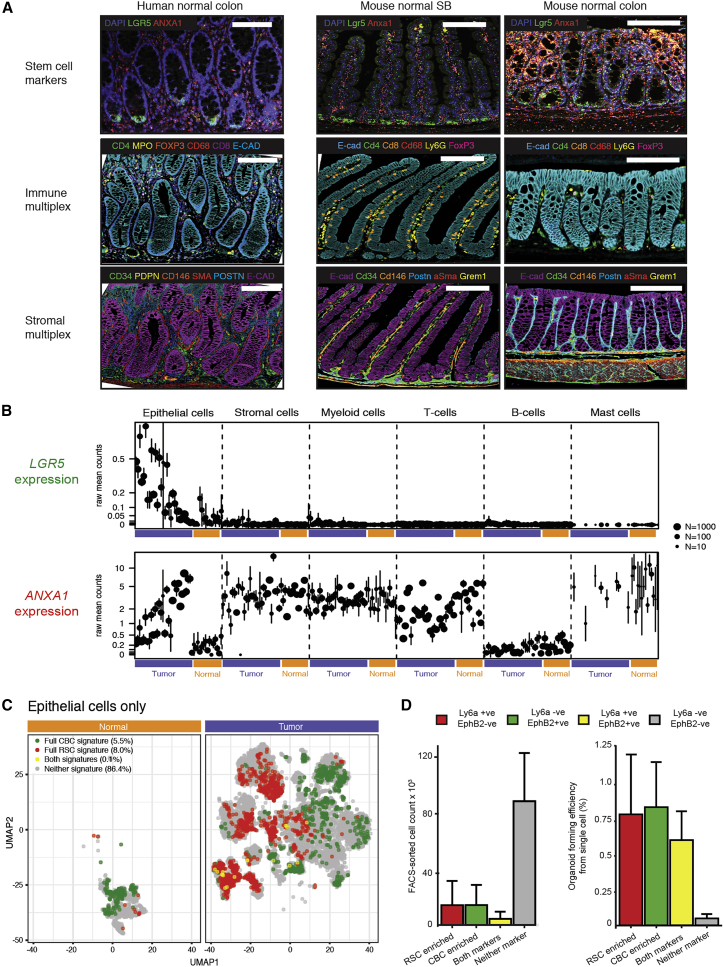
Molecular and morphological assessment of the crypt-base columnar and regenerative stem cell marker expression spectrum (A) Dual color in situ hybridization (ISH) and multiplex immunohistochemistry (IHC) to show expression pattern of representative CBC and RSC markers alongside homeostatic immune and stromal cell distribution in normal mouse and human intestine. Scale bar, 100 μm. (B) Distribution of human multicompartmental scRNA expression of *LGR5* (CBC marker) and *ANXA1* (RSC marker) in normal compartments (orange bars) and cancer cell compartments (purple bars). Mean expression and 95% confidence intervals are shown. (C) Uniform Manifold Approximation and Projection (UMAP) plot of single epithelial cells from human normal and colorectal cancer samples showing cell populations enriched for CBC (green cells), RSC (red cells), and mixed CBC and RSC gene expression (yellow cells). Cells with no enriched stem cell signature expression are shown in gray. (D) Stem cell-marker-expressing cell count and organoid forming efficiency from plated single cells following FACS segregation of KPN mouse primary tumors, measured at day 7 post seeding (mean ± SD shown).

First, we selected an established CBC signature (Munoz et al., 2012) and defined an RSC signature by aggregating published RSC signatures (Mustata et al., 2013; Yui et al., 2018) and refining these based on information on cell-type-associated expression from single-cell RNA-seq data (Methods). Next, we used fluorescent *in situ* hybridization, multiplex immunohistochemistry, and human single-cell RNA expression data to assess normal cell compartment expression of CBC and RSC genes (Figures 1A–1C). In steady state, *LGR5* expression (CBC cell marker) was seen in discrete cell populations and was confined to epithelial cells at the base of the crypts. In contrast, we saw no homeostatic epithelial expression of *ANXA1* (a widely used RSC marker) (Nusse et al., 2018; Yui et al., 2018) in normal human colon or murine small intestine, although low-level expression was seen in the distal colon of mice. However, *ANXA1* expression was detected in stromal, myeloid, and T cells both in human scRNA datasets and on-slide in mouse and human tissue (Figures 1A and 1B). Additional regenerative morphological stem cell markers, *Ly6a* (mouse) and *PLAUR* (human—as there is no human ortholog of *Ly6a*), were also assessed across all lesions (Figure S1).

Assessment of cell-specific expression of *LGR5* and *ANXA1* from scRNA of human cancers revealed an inversely proportional enrichment of these individual cell markers in the cancer epithelial cell compartment, indicating that individual tumors may be variably skewed toward CBC- or RSC-predominant stem cell phenotypes (Figure 1B). Discrete stem cell populations could also be distinguished by application of full CBC and RSC gene profiles in human normal tissue and cancer scRNA datasets, with a small number of cells expressing both stem cell signatures (Figure 1C). Together these data show that despite the cell compartment promiscuity of some RSC markers, application of CBC and RSC expression signatures and morphological markers can be used to identify different epithelial stem cell populations in mouse and human intestinal tissue.

### Stem cell potential of mouse tumor CBC and RSC enriched cell populations

To demonstrate that cells expressing CBC and RSC markers retain stem cell potential in tumors and to test whether CBCs and RSCs occupy comparable fitness peaks, we turned to mouse models of intestinal carcinogenesis to undertake *ex vivo* single-cell clonogenicity experiments. New mouse models cumulatively combine multiple alleles to replicate key CRC epithelial driver gene mutations and generate a range of disease states that phenocopy human polyposis syndromes and the CRC consensus molecular subtypes (Jackstadt et al., 2019). Intestinal cancers from the autochthonous *Vil1-CreER*^*T2*^*;Kras*^*G12D*^*;p53* ^*fl/fl*^*;Rosa26*^*N1ICD/+*^ (KPN) line were digested and cell suspensions were subjected to established protocols for cell segregation and organoid generation, previously used to enrich for CBCs (Merlos-Suarez et al., 2011) and RSCs (Yui et al., 2018) from mouse models. Controlled flow gating strategies (Figure S2) across individual tumors obtained variable numbers of CBC- and RSC-enriched cell populations for each biological repeat, but consistent with human scRNA data (Figure 1C), there were numerically fewer intermediate cells expressing both sets of stem cell surface markers (Figure 1D). Single-cell organoid generation experiments demonstrated that cell populations enriched for CBC, RSC, or both cell markers were capable of single-cell organoid generation, indicating retained stem cell potential, whereas cells without expression of any stem cell marker had very little clonogenic capacity *ex vivo* (Figure 1D).

#### Stem cell marker expression varies across a range of human tumor molecular subtypes and mouse intestinal neoplasia models

Next, we assessed the phenotypic landscape in bulk transcriptome samples from CRC to see if we could infer neoplastic stem cell population admixture using these translationally relevant sample sets. We calculated single-sample enrichment scores for our molecular CBC and RSC molecular signatures using gene set variation analysis (GSVA, Hänzelmann et al., 2013) and derived an intestinal stem cell index measurement by subtracting the CBC score from the RSC score. This allowed assessment of the relative abundance of different stem cell signatures across a wide range of human and mouse tumors.

**Figure 2:**
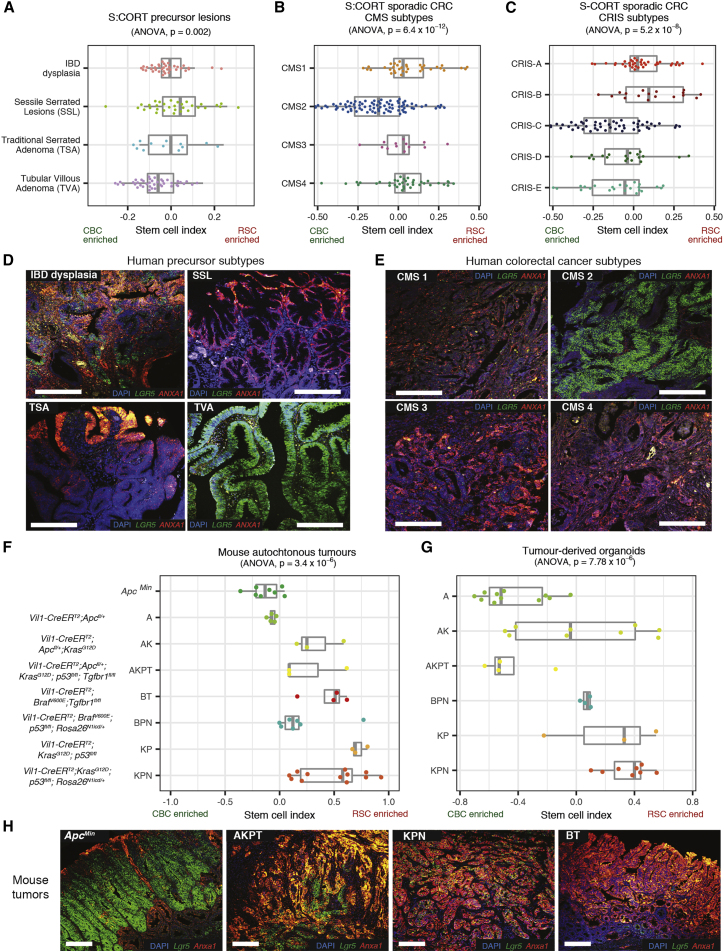
Application of stem cell index to mouse and human neoplasia (A–C) Using stem cell index to map human colorectal precursor lesions (A), colorectal cancer consensus molecular subtypes (CMS) (B), and colorectal cancer intrinsic subtypes (CRIS) (C) across a molecularly defined CBC to RSC expression spectrum (using S-CORT datasets). (D and E) Dual-color ISH for *LGR5* (CBC marker, green) and *ANXA1* (RSC marker, red) expression in representative human precursor lesions (D) and representative human colorectal cancers (E) segregated by consensus molecular subtype. (F and G) Using stem cell index to map mouse autochthonous tumors (F) and matched derived organoids (G) across a molecularly defined CBC to RSC expression spectrum. (H) Dual-color ISH for *Lgr5* (CBC marker, green) and *Anxa1* (RSC marker, red) expression in representative genotype tumors across the CBC to RSC spectrum. Statistical analysis, ANOVA, p values as stated. All animals crossed with *Vil-CreER*^*T2*^. Scale bars, 100 μm. Driver alleles initialization: A is *Apc*^*fl/+*^, *Apc*^*Min*^ is *Apc*^*Min*^, B is *Braf*^*V600E*^, K is *Kras*^*G12D*^, P is *p53*^*fl/fl*^, T is *Tgfβr1*^*fl/fl*^, N is *Rosa26*^*N1icd/+*^.

First, we applied the stem cell index and morphological markers to human polyp and CRC bulk transcriptome data from the S:CORT and Oxford colitis datasets. In both polyps and tumors, we observed variation in expression of stem cell molecular signatures and morphological markers along a phenotypic axis, indicating variable enrichment/admixture of CBC and RSC phenotypes. Individual lesions clustered by histological or molecular subtype across this axis, with CBC predominance in conventional pathway lesions such as tubulovillous adenomas, CMS2, and CRIS-C,D,E cancer subtypes. Conversely, relative enrichment for RSC was seen in serrated precursor lesions and tumor molecular subtypes CMS4 and CRIS-B (Figures 2A–2C). *In situ* hybridization confirmed differential epithelial marker *LGR5* and *ANXA1/PLAUR* expression across representative lesions (Figures 2D, 2E, and S1).

Next, we applied the mouse version of the stem cell index to murine bulk transcriptome data. Mouse models permit control over the epithelial genotype, which allowed assessment of the cumulative impact of individual driver genes on molecular phenotype in both autochthonous tumors and derived organoids. Similar to what is seen in human lesions, we observed variation in the stem cell index across a CBC to RSC phenotypic axis, with individual lesions clustering by mouse epithelial genotype (Figure 2F). Transgenic manipulation of some key drivers produced a notable skew in cell admixture. Thus, epithelial *Apc* mutation enriched for CBC cell marker expression, whereas Mapk activation (through *Kras* or *Braf* mutation) or Tgfβ disruption (through *Tgfβr1* knockout) skewed the stem cell index toward RSC markers. In multi-allele models, the cumulative accumulation of driver genes correlated with variable degrees of admixture of stem cell marker expression. In mouse cancer organoids, the baseline stem cell index was distinct for each genotype and the pattern of stem cell index distribution across different genotypes was conserved between the *ex vivo* bulk tumor transcriptome and the derived *in vitro* organoids, cultured in the absence of other niche cellular constituents (Figure 2G). This indicates the predominant contribution of changes in epithelial gene expression to the variance in the stem cell index. Multicolor *in situ* hybridization was used to confirm variable epithelial expression of CBC and RSC markers in four key disease positioned animal models: *Apc*^*Min*^, AKPT, KPN, and BT (Figure 2H). These models were selected to represent conventional and serrated molecular carcinogenesis pathways and span the stem cell phenotypic axis (Jackstadt et al., 2019; Lannagan et al., 2021; Leach et al., 2021). Together these data demonstrate that the stem cell index can be used to assess variable admixture of stem cell phenotypes across a range of mouse and human tumors.

### Driver genes and pathways associated with variable stem cell molecular phenotype

**Figure 3:**
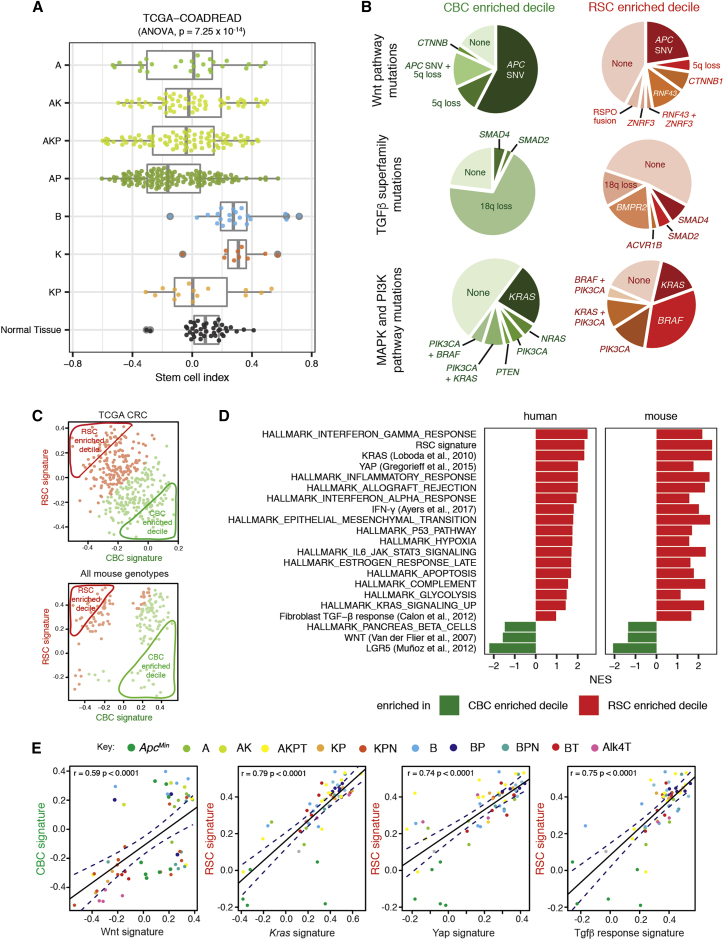
Driver genes and pathways associated with variable stem cell molecular phenotype (A) Human genotype-stem cell phenotype correlation based on stem cell index distribution in TCGA tumors with different putative driver gene single-nucleotide variant (SNV) mutation genotypes, contrasted to normal tissue from same dataset (driver gene initials: A is APC, K is KRAS, P is p53, B is BRAF). (B) Comparison of mutation type and prevalence disrupting the Wnt pathway, MAPK and PIK3CA pathways, and the TGFβ superfamily in TCGA tumors subdivided into CBC- and RSC-predominant deciles. (C) Segregation of mouse and human lesions by CBC (x axis) and RSC (y axis) signature expression. Predominant (above median) expression signature in each tumor is defined by color (CBC in green and RSC in red), and the 10% most polarized CBC- or RSC-expressing tumors were segregated into CBC- and RSC-enriched deciles for comparison. (D) Gene set enrichment analysis of hallmark and select pathways in bulk transcriptome from human tumors (TCGA) and murine lesions (Glasgow dataset) segregated into CBC- and RSC-predominant deciles. Pathways shown have *P*_FDR_ ≤ 0.25 apart from YAP in the mouse lesions and Fibroblast TGFβ response in the human tumors. (E) Correlation of key pathway expression signatures with CBC or RSC gene expression across a range of mouse models. Different genotypes are identified by different colors as determined by the key. Driver alleles initialization: A is *Apc*^*fl/+*^, *Apc*^*Min*^ is *Apc*^*Min*^, B is *Braf*^*V600E*^, K is *Kras*^*G12D*^, P is *p53*^*fl/fl*^, T is *Tgfβr1*^*fl/fl*^, N is *Rosa26*^*N1icd/+*^, Alk4 is *Alk4*^*fl/fl*^.

To assess human tumor genotype-stem cell phenotype correlations, we looked for associations of key driver gene mutations with stem cell phenotype in published human single-cell datasets (Lee et al., 2020). We found significant correlation between epithelial *APC* and *BRAF* mutations with single-cell transcriptome-derived CBC and RSC phenotypes, respectively. However, there was no association with other key drivers such as *p53* and *KRAS*, which were seen in cells across the phenotypic spectrum (Figure S3A). Next, we mapped the distribution of the stem cell index in TCGA lesions stratified by key CRC driver mutations to see if the stem cell index variation mirrored that seen with the clearly defined mouse genotypes (Figure 3A). We then segregated TCGA tumors into polarized deciles for stem cell marker expression (Figure 3C) and undertook pairwise comparison of single nucleotide variation (Figure S3B) and copy number variation (Figure S3C) between CBC and RSC predominant tumors. In the tumors most enriched for CBCs, ligand-independent Wnt mutations (*APC* and *CTNNB1*) were found in 84% of tumors but only 35% of RS- predominant lesions, where ligand-dependent Wnt alterations such as *RNF43*, *ZNRF3*, and *RSPO2/3* fusions were also seen (Figure 3B). There was also variation in the mutation prevalence and type impacting the MAPK/PI3K pathways and the TGFβ superfamily (Figure 3B). Together, these data indicate an association between optimally selected driver gene mutations in key signaling pathways and the predominant stem cell phenotype in human lesions.

Next, we used transcriptome data from the TCGA and our suite of animal models to compare cross-species signaling pathway disruption. Mouse and human tumors were segregated by stem cell index, and gene set enrichment analysis was used to contrast hallmark pathway disruption in the most CBC- and RSC-predominant deciles. Strikingly, we saw significant cross-species correlation between stem cell phenotype and signaling pathway disruption, with activation of Wnt signaling in CBC-predominant lesions and enrichment of KRAS, YAP, TGFβ, and inflammatory pathways (such as IFN-γ) in RSC-enriched mouse and human tumors (Figures 3C and 3D). In mouse models, we mapped expression of these key pathways with CBC or RSC gene signatures across a large range of defined genotypes (Figure 3E). Although we identify some key cross-species candidate signaling hubs, we also show that there are a number of genotype-specific pathways associated with a predominant CBC or RSC phenotype across three of our key mouse genotypes (*Apc*^*Min*^, KPN, and AKPT, Figure S3D, associated pathways listed in Table S2). Thus, it seems likely that individual tumors utilize both shared and genotype/tumor-specific cell-intrinsic and extrinsic pathways to establish phenotypically convergent stem cell populations.

**Figure 4:**
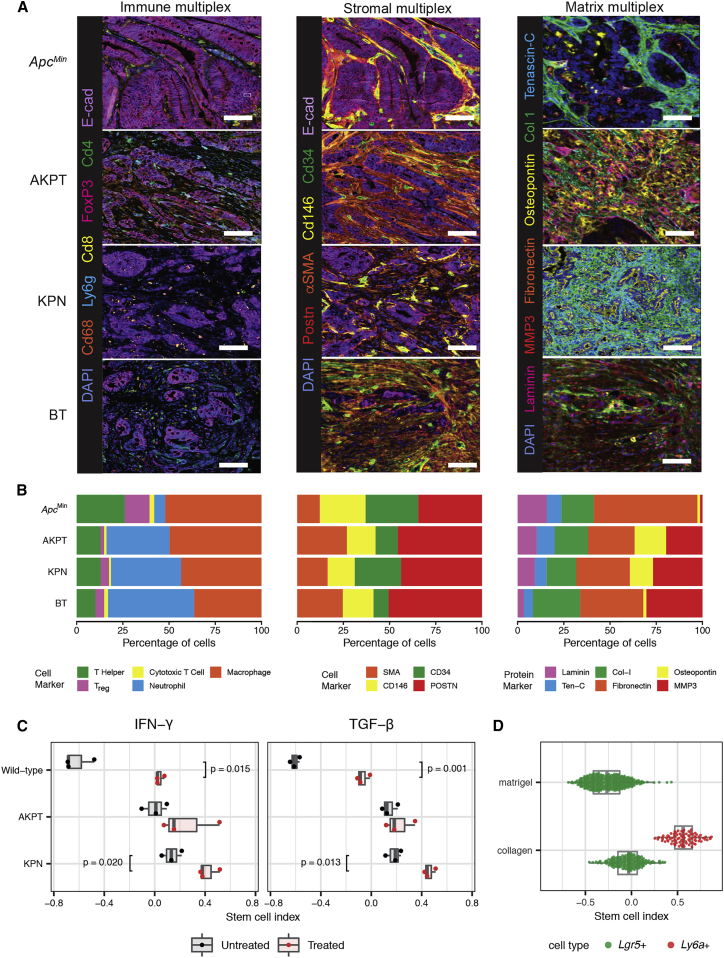
Microenvironmental landscaping and crosstalk influences epithelial stem cell phenotype (A) Representative multiplex IHC images of immune, stromal, and matrix landscapes in mouse tumors selected from across the stem cell phenotypic axis. Scale bars, 100 μm. (B) Variable proportion of different cell/matrix components in tumors from each genotype quantified from multiplex IHC images (n = 3 mice per genotype). (C) Impact of media supplementation of IFN-γ (1 μL/mL) and TGFβ1 (0.5 μL/mL) on stem cell phenotype of wild-type and AKPT and KPN mouse tumor organoids. t test, p values as stated. (D) Stem cell index applied to single-cell transcriptome data derived from organoids grown in Matrigel or collagen matrix (from Ramadan et al., 2021).

Together these data imply that epithelial ligand-independent Wnt signaling mutation (such as *APC*) enhances fitness of the CBC stem cell phenotype, whereas RSC fitness may be influenced by signaling disruption from both epithelial cell-intrinsic (e.g. *KRAS* and *BRAF*) and tumor microenvironmental sources, with some key pro-regenerative stem cell pathways mapping predominantly to immune (IFN-γ), stromal (TGFβ), and matrix (YAP) cell compartments. In light of this observation, we used multiplex staining to assess the cell compartment landscape of representative mouse tumors from our four key genotypes selected to span across the CBC to RSC phenotypic axis. We found quantifiable differences in the immune, stromal, and matrix landscapes in tumors from different models (Figures 4A and 4B), with the matrix compartment in particular showing both interesting intra-tumor topographic heterogeneity (Figure S4A) and inter-tumor diversity between lesions from different genotypes (assessed using the Shannon index, Figure S4B). This was consistent with differential landscaping of the tumor context in the lesions from each of the different models, generating variable microenvironmental niches. We hypothesized that niche crosstalk back to the epithelium (through secreted signaling or mechano-transduction) could influence epithelial stem cell phenotype. To test the effect of secreted signaling directly, we turned to mouse and human tumor organoids and used cytokine/morphogen supplementation of the media to model the influence of immune and stromal cell signaling, respectively. We saw a significant shift in the stem cell index toward an RSC-enriched phenotype in normal organoids following media supplementation of both IFN-γ and TGFβ (Figure 4C). In mouse cancer organoids, the genotype-specific stem cell phenotype was not fixed: we saw similar directional shifts in the stem cell index in the response of KPN mouse cancer organoids to both IFN-γ and TGFβ. However, AKPT organoids only responded to IFN-γ, consistent with the knockout of the *Tgfβr1* receptor in this genotype (Figure 4C). This shows that manipulation of immune-derived and stromally derived signaling pathways can directly modulate epithelial phenotype. Furthermore, although the baseline stem cell phenotypic state is defined by organoid genotype (Figure 2G), it is not completely fixed by cancer cell driver mutation alone and remains at least partly responsive to microenvironmental signaling.

Constitutive YAP activation, through *Lats1/2* kinase knockout, induces fetal signature gene expression in murine organoids (Cheung et al., 2020). We (Koppens et al., 2021; Ramadan et al., 2021) and others (Yui et al., 2018) have shown that organoid growth in collagen can induce YAP and model the impact of matrix mechano-transduction (Figures S4C–S4E). To assess the effect of YAP activation on stem cell phenotype, we analyzed single-cell data from organoids grown in collagen and Matrigel from Ramadan et al. (2021). We see the emergence of a *Ly6a*+ cell population only in organoids maintained in collagen, driving a significant shift in the stem cell index toward the regenerative phenotype (Figure 4D). This shift was confirmed in two independent bulk organoid transcriptome datasets, ArrayExpress: E-MTAB-5247 (Yui et al., 2018, Figure S4D) and ArrayExpress: E-MTAB-10082 (Ramadan et al., 2021, Figure S4E).

Together these data demonstrate that variance in the stem cell molecular phenotype is associated with differences in epithelial cell-intrinsic driver gene mutations alongside conserved cross-species disruption of key microenvironmental signaling pathways. Intercompartmental crosstalk from variably landscaped immune, stromal, and matrix compartments across different tumors can directly influence epithelial stem cell phenotype through secreted signaling or mechano-transduction pathways.

### A dynamic equilibrium exists between neoplastic-stem-cell-marker-expressing populations

**Figure 5:**
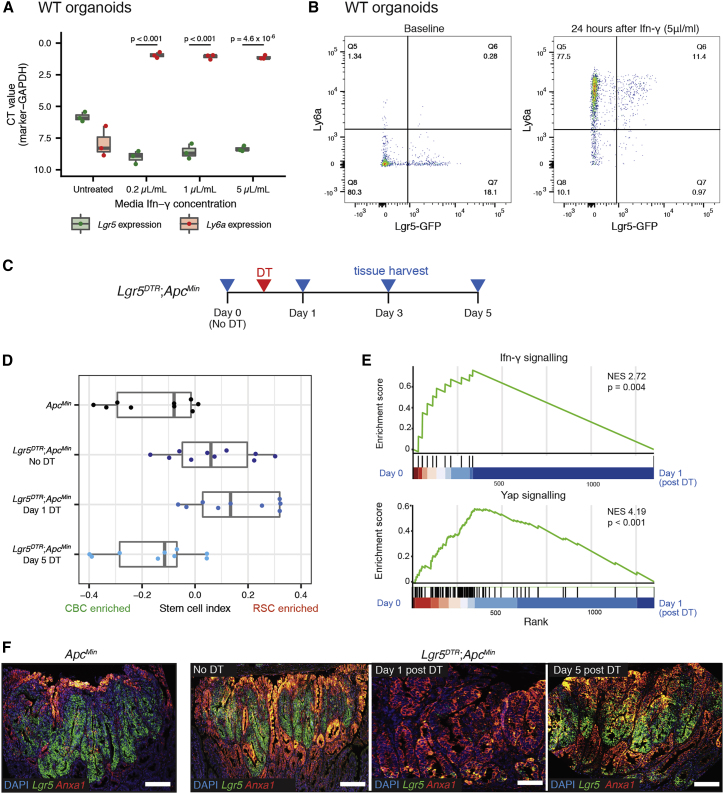
Adaptive shift of stem cell phenotype under selective pressure (A) Shift in stem cell-marker expression detected by qRT-PCR following exposure of wild-type organoids to increasing concentrations of media IFN-γ. Statistical analysis, t test, p values as stated. (B) Skewed expression of stem cell markers, detected by FACS for Ly6a and GFP, following exposure of Lgr5-GFP labeled murine organoids to 5 μL/mL of media IFN-γ. (C) Schematic showing timing of recombination, Diphtheria Toxin (DT) activation, and tissue harvesting of *Lgr5*^*DTR*^*;Apc*^*Min*^ mice. (D) Using stem cell index to map polyp tissue from *Apc*^*Min*^ and *Lgr5*^*DTR*^*;Apc*^*Min*^ to show dynamic change in stem cell molecular phenotype measured by stem cell index, before and after DTR activation and CBC cell ablation. (E) Gene set enrichment analysis showing enrichment of Ifn-γ signaling and Yap signaling between day 0 (unrecombined) and day 1 (after DTR stem cell ablation). (F) Dual-color ISH for *Lgr5* (CBC marker, green) and *Anxa1* (RSC marker, red) to show marker expression change in *Apc*^*Min*^ and *Lgr5*^*DTR*^*;Apc*^*Min*^ polyps before and after CBC ablation. Scale bars, 100 μm.

From our molecular analysis of large tumor sets, it was evident that expression of stem cell markers ranged across a CBC to RSC phenotypic axis, with single-cell assessment, FACS segregation, and morphological assessment of different lesions all demonstrating the co-existence of discrete CBC, RSC, and shared marker-expressing cell populations in mouse and human tumors. Following injury, RSCs are capable of reconstituting lost CBC cell populations (reviewed in de Sousa and de Sauvage, 2019), so we reasoned that the dysregulated signaling of the tumor milieu permitted a dynamic stem cell equilibrium with adaptive interconversion between cells situated on CBC and RSC phenotype peaks. To test this *in vitro*, we identified a dose of media IFN-γ that induced the most significant shift in stem cell phenotype in organoids grown in Matrigel (Figure 5A). We then cultured Lgr5-GFP mouse organoids to permit direct flow cytometric detection of an *Lgr5*+ve stem cell population and used high-dose media IFN-γ to apply a selective pressure to GFP-labeled cells. Flow cytometry analysis showed that IFN-γ media supplementation rapidly skewed cell phenotype, with all organoid cells including both GFP-high expressing CBC cells and GFP-low expressing progenitor cells profoundly upregulating *Ly6a* expression, consistent with a plastic adaptive shift along the CBC to RSC phenotypic axis (Figure 5B).

To test whether this adaptive response to selective pressure could occur *in vivo,* we crossed *Apc*^*Min*^ animals with the *Lgr5*^*DTR*^ allele established by Tian et al. (2011) to generate *Lgr5*^*DTR*^*;Apc*^*Min*^ animals. As previously noted (Tan et al., 2021), targeted introduction of the DTR cassette and *Lgr5* hemizygosity impacted *Lgr5*+ve CBC number in *Lgr5*^*DTR*^*;Apc*^*Min*^ mouse polyps at steady state (Figure S5A) and in polyps (Figure S5B) and resulted in a detectable shift in the stem cell index in comparison with *Apc*^*Min*^ lesions alone (Figure 5D). Following diptheria toxin injection, almost all *Lgr5*+ve cells in established *Lgr5*^*DTR*^*;Apc*^*Min*^ mouse polyps were selectively ablated, and we tracked the molecular phenotypic response to acute loss of the predominant stem cell population (Figures 5D–5F). After 24 h, ablation of *Lgr5*+ve cells provoked a dramatic upregulation of RSCs in *Lgr5*^*DTR*^*;Apc*^*Min*^ polyps, followed by partial reconstitution and recovery of *Lgr5*-expressing CBCs by day 5 (Figures 5D and 5F). These shifts in the stem cell molecular phenotype were associated with acute upregulation of Ifn-γ, Yap (Figure 5E), and Kras signaling pathways (Figure S5C), but notably, there was no significant impact on polyp size, cell proliferation, or apoptotic cell death following recombination (Figures S5E and S5F). This indicates that following selective ablation, rapid adaptive shifts in tumor signaling act to rapidly restore the stem cell equilibrium and that these profound shifts in stem cell phenotype can occur without detectable change in conventional tumor clinical response measurements.

#### Temporally spaced assessment of stem cell index can be used to assess adaptive response to selective pressure

**Figure 6:**
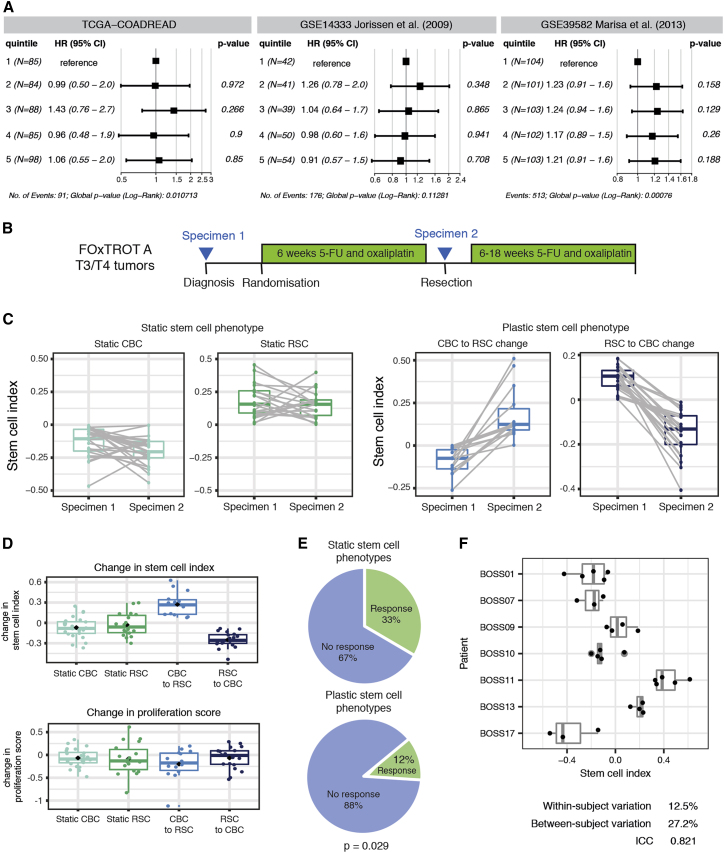
Human translational implications (A) Forest plots of progression-free survival (PFS) Hazard Ratios (HRs) (TCGA-COADREAD) and disease-free survival (DFS) HRs (Jorissen et al., 2009; Marisa et al., 2013) for quintiles of tumor stem cell index. Data are presented as HR with error bars indicating the 95% confidence interval (CI). p values from a Cox proportional hazards regression are shown. (B) FOxTROT (track A) trial schedule showing specimen acquisition before (specimen 1) and after 6 weeks (specimen 2) of 5-FU and oxaliplatin chemotherapy. (C) Ladder plots showing GSVA (RSC-CBC signature) of human tumor samples before and after chemotherapy, with patients grouped as “static” or “plastic” depending on magnitude of signature change following therapy. (D) No change in cell proliferation score in groups of tumors segregated by post treatment shift in stem cell index. (E) Proportion of patients with a documented response to chemotherapy in the FOxTROT trial when grouped by static or plastic stem cell response to treatment. Statistical analysis, Fisher’s exact test, p value as stated. (F) Low within-subject variation of the stem cell index from random non-adjacent biopsies from the BOSS trial.

As part of a fitness landscape model, we propose that interlinked CBC and RSC population peaks co-exist in CRC and that in untreated tumors, different combinations of epithelial mutation and microenvironmental signaling shift the stem cell population distribution to an equilibrium set point that varies between individual lesions. Given that there is some clustering of individual lesion set points within the established human CRC molecular subtypes (Figures 2B and 2C), we assessed whether snapshot measurement of individual tumor stem cell index was associated with clinical outcome. We did not observe any significant associations between quintiles of tumor stem cell index and survival in three CRC datasets after a Cox proportional hazards regression, indicating that snapshot measurement of stem cell index is not as informative as a reliable, standalone prognostic molecular signature. However, in mouse and organoid models, temporally spaced assessment of the stem cell index did detect dynamic and adaptive shifts in the tumor molecular phenotype in response to acute selective pressures. To undertake comparable assessment of molecular phenotypic shifts in human colorectal cancer, we turned to the Fluoropyrimidine, Oxaliplatin & Targeted Receptor pre-Operative Therapy for colon cancer (FOxTROT) trial dataset (track A). This unique cohort of patients were randomized to 6 weeks neoadjuvant oxaliplatin and 5-FU treatment between diagnostic biopsy and surgical resection, enabling temporally spaced transcriptional analysis of the tumor in response to a therapeutic selective pressure (Figure 6B) (Seymour et al., 2019). A spectrum of response to therapy could be identified, ranging between negligible and dynamic shifts in the stem cell index, and patients were grouped into “static” or “plastic” groups, respectively (Figure 6C). Patients with “plastic” stem cell phenotype had no correlative change in transcriptome-based cell proliferation score (Figure 6D) but were significantly less likely to have a histologically detectable response to chemotherapy, indicating an association between the tumor capacity for adaptive change and clinical response to treatment (Figure 6E).

We considered that intra-tumor heterogeneity with resultant variability between biopsy and resection specimens could be a key potential confounder when interpreting consecutive samples in FOxTROT. It has been noted that sample heterogeneity contributes to highly variable consensus molecular subtype assignment in multiregional biopsy samples (Ubink et al., 2017), and this has been attributed to different proportions of stromal and immune cells in different biopsies (Alderdice et al., 2018). To assess this, we analyzed stem cell index in the transcriptome data derived from non-adjacent, multiregional biopsies in the Biopsies of Surgical Specimens (BOSS) study (Ubink et al., 2017) and found a relatively high intra-class correlation coefficient (ICC = 0.821, Figure 6F). The within-subject coefficient of variation (variation between random biopsies from the same tumor, 12.5%) was lower than the between-subject coefficient of variation (27.2%), indicating that measurement of the stem cell index can transcend sample “contamination” with non-epithelial tissue. Thus we concluded that measurement of the stem cell index from biopsy samples produces a reasonably robust representation of the stem cell phenotype of the whole tumor, which is reassuring for the conclusions drawn from temporally assessed samples in FOxTROT.

Together these data indicate that CBC and RSC marker-expressing cell populations can co-exist in intestinal neoplastic lesions and that plastic cells can adaptively shift between these stem cell phenotypes in response to transgenic ablative or therapeutic selective pressures. Importantly, it is the capacity of the tumor to adapt, assessed through a responsive shift in the stem cell phenotype, rather than a snapshot assessment of the pre-treatment phenotypic equilibrium set point that is associated with chemo-responsiveness.

## Discussion

In the injured intestine, cell dedifferentiation is an evolved, physiological response to enable rapid epithelial regeneration through temporary disruption of morphogen signaling and relaxation of stringent homeostatic controls over cell fate. Despite the neoplastic co-option and corruption of the same conserved signaling pathways, the contribution of analogous stem cell plasticity in colorectal tumors has not been fully established. This has clinical relevance, as cellular plasticity and resultant phenotypic heterogeneity drives therapy evasion, which has predominantly been noted in tumors where there is a definable morphological phenotypic switch—such as neuroendocrine differentiation in castration-resistant prostate cancer and small cell conversion in treated non-small cell lung cancer (Boumahdi and de Sauvage, 2020). Assessment of this phenomenon in colorectal cancer has been hampered by the lack of a defined, measurable, and pathologically relevant phenotypic state that can temporally track lineage fate.

Here, we have used a combined molecular and morphological approach to assess stem cell phenotypic heterogeneity across a wide range of mouse and human intestinal tumors and have developed an applicable and accessible tool to measure it— the stem cell index. This tool can be deployed on bulk tumor samples and is capable of detecting variable enrichment and admixture of CBC and RSC transcriptional signatures across lesions. This indicates that different populations of stem cells can co-exist in both benign and malignant neoplastic settings. Although not all cells actively expressing established stem cell markers necessarily serve as functioning stem cells (Barriga et al., 2017; Kozar et al., 2013), we were able to demonstrate comparable *ex vivo* stem cell potential in disaggregated primary tumor cell populations following FACS enrichment for CBC and RSC markers. Directional skewing of phenotype toward CBC or RSC predominance correlates with specific driver mutation(s) and/or disruption of microenvironmental signaling, with remarkable conservation of identified key pathways across mouse and human tumors. Using mouse models to control for epithelial mutation, we have generated data to support a co-evolutionary, crosstalk model of solid tumor carcinogenesis, where epithelial accumulation of somatic mutation results in remodeling of the tumor context to establish genotype-distinct niches, comprising variable immune, stromal, and matrix components. Key morphogen pathways associated with these distinct microenvironmental cell compartments then signal back to the epithelium to induce cell plasticity and adaptively regulate stem cell phenotype. Although we show correlation of different stem cell phenotypes with some key signaling pathways, and we note the overlap with the mechanisms that regulate murine organoid Wnt independence (Han et al., 2020) and the induction of a chemoresistant, quiescent population in patient-derived organoids (Sole et al., 2022), it is likely that multiple convergent and redundant mechanisms are involved in the adaptive response to distinct selective pressures. We believe that the impact of mouse genotype on variable microenvironmental remodeling (Figure 4A) means that different tumors may well utilize different cell-intrinsic and extrinsic strategies to enable stem cell plasticity. This needs to be tested comprehensively and systematically in order to establish key shared and genotype-specific druggable signaling hubs and identify any important functional redundancies. Further detailed work, using advanced biological models, is needed to identify, map, and therapeutically manipulate the combinatory networks of signaling disruption that induce, regulate, and constrain stem cell plasticity in different tumor settings.

Consistent with a role for bidirectional epithelial-microenvironmental crosstalk in regulating stem cell phenotype, we use the stem cell index to demonstrate rapid and measurable adaptive change in response to media manipulation of microenvironmental signaling *in vitro* or an ablative direct selective pressure to CBCs *in vivo*. Loss of one stem cell population *in vivo* provokes cell plasticity and an acute adaptive switch in phenotype, with subsequent rapid recovery and restitution of the ablated CBC phenotype, restoring an equilibrated heterogeneic stem cell population. This is consistent with data from recently published models of secondary tumors. Circulating metastatic cells were noted to be predominantly *Lgr5*−ve; however, cell plasticity with *Lgr5*+ve stem cell reconstitution in the liver was required for secondary outgrowth (Fumagalli et al., 2020). Furthermore, constitutive YAP activation, with resultant continuous drive toward an RSC phenotype, prevented re-establishment of a stem cell equilibrium and abrogated orthotopic xenograft tumor or metastatic outgrowth (Cheung et al., 2020; Heinz et al., 2022). Together, this indicates the importance of a heterogeneous and dynamic stem cell population to enable adaptive response to selective pressures and to facilitate lesion outgrowth.

Here, in primary tumors, we demonstrate restoration of the stem cell equilibrium within 5 days of selective ablation, through rapid induction of cell plasticity and adaptive phenotypic shift. Deployment of the stem cell index was able to detect this profound change in the stem cell phenotype, but interestingly, mean lesion size in the animals was unaffected, and cell proliferation continued unchecked. A comparable dynamic change was detected in a proportion of patients in the valuable FOxTROT cohort, where serial sampling permits assessment of temporal change under the influence of a chemotherapeutic selective pressure. In this unique trial cohort, the use of temporally spaced stem cell index assessments acts as a measure of tumor adaption and demonstrates that the capacity for dynamic change in stem cell marker expression was associated with reduced histological response to therapy. These combined preclinical and human data demonstrate that induced shifts in the molecular phenotype may occur independently of the response seen using conventional assessments of clinical disease activity, such as tumor size or cell proliferation. Stem cell molecular phenotype may be an informative metric that is currently going unmeasured when considering patient response to therapy. The capacity to temporally track cell fate and potentially titrate treatments, according to detectable changes in a disease-relevant molecular phenotype, could become a powerful tool in patients undergoing neoadjuvant therapies, such as chemoradiotherapy in rectal cancer.

**Figure 7:**
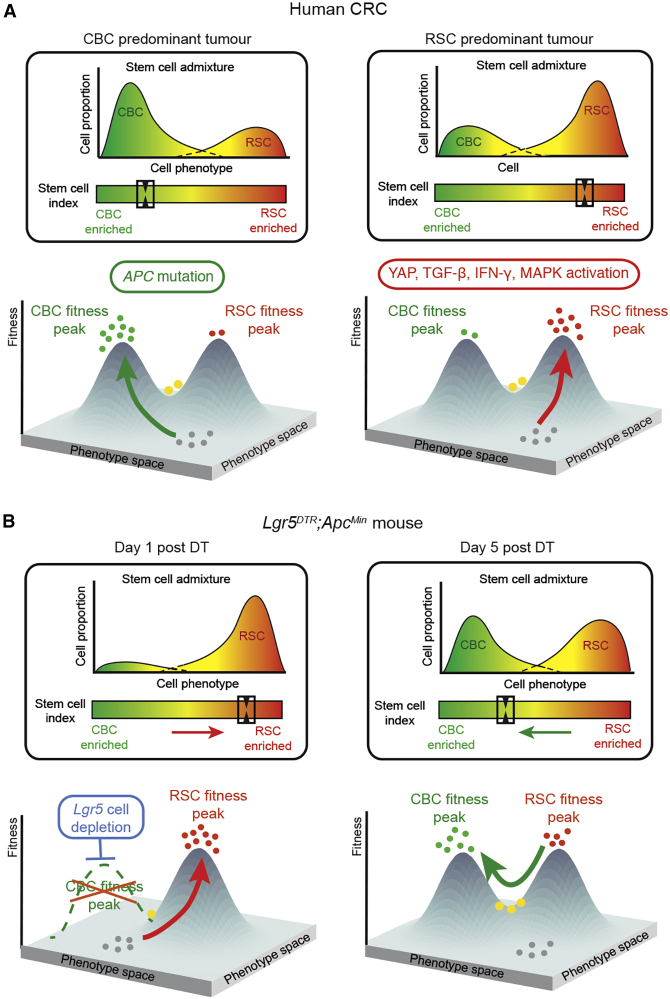
Fitness landscape model Tumor stem cell phenotype can be represented as a fitness landscape model, where *Lgr5*+ve CBC and *Lgr5*−ve RSC represent distinct but interlinked fitness peaks situated along a phenotypic axis. (A) Epithelial cells “climb” these fitness peaks (arrows) through the combination of acquired epithelial mutations and the influence of microenvironmental signaling, placing them at distinct points within the fitness landscape. Bulk transcriptome data can be used to calculate the stem cell index, which reflects stem cell population admixture and can be used as a measure of individual tumor position within this phenotypic axis (boxes). (B) Application of a selective pressure to a fitness peak (e.g *Lgr5+* CBC ablation) alters the morphogenic signaling landscape and shifts the stem cell equilibrium toward an alternative phenotype at day 1. Rapid regeneration of the lost CBC population restores the stem cell equilibrium after 5 days. These dynamic shifts can be measured by change in the stem cell index (boxes). Key: green dots, CBC cells; red dots, RSC cells; yellow dots, both marker-expressing cells; gray dots, no stem cell-marker-expressing cells.

Using the data presented here we propose a modified fitness landscape model of stem cell phenotype in colorectal cancer, where *Lgr5*+ve CBCs and *Lgr5*−ve RSCs represent distinct but interlinked and equilibrated stem cell population peaks situated along a phenotypic axis. Epithelial cells shift their stem cell phenotype along this axis through the combination of acquired epithelial mutation and the influence of microenvironmental signaling (Figure 7A), which arises from surrounding niches composed of variably remodeled immune, stromal, and matrix tissue compartments. Establishment of a tumor-specific stem cell population equilibrium depends on the balance of cell-intrinsic and extrinsic signaling. Application of a selective pressure to a stem cell phenotype peak alters microenvironmental morphogenic signaling, resulting in a new fitness landscape, and temporarily shifts the stem cell population distribution toward an alternative phenotype. This cell plasticity allows reconstitution of the lost stem cell population, facilitating a recovery of the stem cell distribution to a new, post-treatment equilibrium (Figure 7B). Critically, it seems to be the capacity of the tumor to enable this adaptive phenotypic shift and recover an equilibrated heterogeneic stem cell population that is associated with chemoresistance, rather than the pre-treatment position of the phenotypic equilibrium set point. This is consistent with recently published work that shows that constitutive YAP activation drives a polarized RSC phenotype and prevents restoration of the dynamic and heterogeneous stem cell population required for lesion outgrowth (Cheung et al., 2020; Heinz et al., 2022). In our model, this can be represented by skewing of the stem cell phenotype onto a single population peak, which constitutes an evolutionary dead end.

We have developed a simple and applicable tool to assess stem cell admixture and detect responsive shifts in the stem cell molecular phenotype. We believe that this can be used to biologically steer neoadjuvant therapies to inform the scheduling of our current therapeutic armamentarium. Concomitant development of new treatments is needed, both to target stem cell populations directly and to close off the emergent adaptive signaling pathways that enable stem cell plasticity in order to abrogate the evolution of therapy resistance.

### Limitations of the study

We have not undertaken whole-exome somatic mutation screening of all of the lesions from the autochthonous animal models, thus it is conceivable that some of the stem cell index intra-genotype variation seen (particularly in the KPN group) is the consequence of undetermined additional somatic mutation. Furthermore, the complex multi-allele mouse models used here currently result in simultaneous genetic recombination. Thus, changes seen in the genotype-specific tumor landscape remodeling are the result of concomitant, combined driver gene mutation rather than the cumulative, stepwise progression seen in human disease.

## STAR★Methods

### Key resources table

**Table:** 

REAGENT or RESOURCE	SOURCE	IDENTIFIER
**Antibodies**		

Human/mouse gremlin antibody	R&D	Cat#AF956
Recombinant Anti-CD34 antibody [EP373Y]	Abcam	Cat#ab81289
Recombinant Anti-CD146 antibody [EPR3208]	Abcam	Cat#ab75769
Anti-alpha smooth muscle Actin antibody	Abcam	Cat#ab5694
Recombinant Anti-Periostin antibody [EPR20806]	Abcam	Cat#ab227049
E-Cadherin (24E10) Rabbit mAb	Cell Signaling Technology	Cat#3195
Purified Rat Anti-Mouse Ly-6G Clone 1A8	BD Biosciences	Cat#551459
Recombinant Anti-CD4 antibody [EPR19514]	Abcam	Cat#ab183685
CD8α (D4W2Z) XP Rabbit mAb	Cell Signaling Technology	Cat#98941
Anti-CD68 antibody	Abcam	Cat#ab125212
FoxP3 (D6O8R) Rabbit mAb	Cell Signaling Technology	Cat#126553
Ki-67 (D3B5) Rabbit mAb	Cell Signaling Technology	Cat#CS12202S
Human/Mouse Active Caspase-3 Antibody	R&D Systems	Cat#AF835
L/D Fixable Green dye	Thermofisher	L34969
Pacific Blue anti-mouse Ly-6A/E (Sca-1) Antibody (4 in 200 uL)	BioLegend	108119
APC-secondary antibody (10 uL in 1 million cells)	RND	F0108
CD31/cy7 (1 in 200 uL)	eBioscience	25-0311-82
CD45/cy7 (0.25 in 200 uL)	PharminGen	561868
Ephb2 primary (0.5 ug/10uL in 1 million cells)	RND	AF467
EpCAM (1 in 200uL)	BD Biosciences	740281
Fc Block	BD Biosciences	553141
anti-mouse Ly6a-APC	eBioscience	#17-5981-81

**Biological Samples**		

Human colorectal polyps and tumors from the S:CORT cohort	S:CORT	N/A
Human colorectal tumors from the Oxford BRC Cancer cohort	Oxford BRC Cancer cohort	N/A
Human colitis tissue samples from the Oxford GI Biobank	Oxford GI Biobank	N/A
Human colorectal polyps from the Oxford GI Biobank	Oxford GI Biobank	N/A

**Chemicals, Peptides, and Recombinant Proteins**		

Tamoxifen	Sigma-Aldrich	Cat# T5648
Neutral buffered formalin	Merck	Cat# HT501128-4L
Diethyl pyrocarbonate (DEPC)	Merck	Cat# D5758-100ML
Diphtheria Toxin (DT)	Merck	Cat # 322326-1mg
IFN-γ recombinant mouse protein	Peprotech	Cat # 315-05
IFN-γ recombinant mouse protein	R&D Systems	Cat # 485-MI
TGFβ recombinant mouse protein	R&D Systems	Cat # 7666-MB

**Critical Commercial Assays**		

High Pure FFPE RNA Isolation kit	Roche Life Sciences	Cat#06650775001
RNeasy Micro kit	Qiagen	Cat#74004
DNA-*free*™ DNA Removal Kit	ThermoFisher	Cat#AM1906
Qubit RNA HS Assay Kit	ThermoFisher	Cat#Q32855
Qubit RNA BR Assay Kit	ThermoFisher	Cat#Q10211
QuantSeq 3′ mRNA-Seq Library Prep Kit FWD	Lexogen	Cat#015.96
High Sensitivity D1000 ScreenTape devices	Agilent	Cat#5067-5584
RNAscope Multiplex Fluorescent Detection Kit v2	Bio-techne	Cat#323110
ISH probe: Mm-Anxa1	ACD Bio (Bio-techne)	Cat#509291
ISH probe: Hs-ANXA1	ACD Bio (Bio-techne)	Cat#465319
ISH probe: Mm-Ly6a-C2	ACD Bio (Bio-techne)	Cat#427571-C2
ISH probe: Hs-PLAUR	ACD Bio (Bio-techne)	Cat#542701
ISH probe: Mm-Clu-C3	ACD Bio (Bio-techne)	Cat#427891-C3
ISH probe: Hs-CLU	ACD Bio (Bio-techne)	Cat#606241

**Deposited Data**		

RNA-seq data from the *Lgr5*^*DTR*^*;Apc*^*Min*^ mouse experiment	This paper	ArrayExpress: E-MTAB-10470.
RNA-seq data from treated intestinal organoids	This paper	ArrayExpress: E-MTAB-11769 ArrayExpress: E-MTAB-11784

**Experimental Models: Cell Lines**		

*villin*Cre^ER^; *Kras*^G12D/+^; Tr*p53*^fl/fl^*Rosa26*^N1icd/+^ (KPN organoids)	Jackstadt et al., (2019)	N/A
*villin*Cre^ER^; *Kras*^G12D/+^; Tr*p53*^fl/fl^ (KP organoids)	Jackstadt et al., (2019)	N/A
*villin*Cre^ER^; *Apc*^fl/fl^; *Kras*^G12D/^+; *Trp53*^fl/fl^*TgfbrI*^fl/fl^ (AKPT organoids)	Jackstadt et al., (2019)	N/A
wild-type C57BL/6J (WT organoids)	This study	N/A

**Experimental Models: Organisms/Strains**		

*Mouse: villin*Cre^ER^; *Kras*^G12D/+^; Tr*p53*^fl/fl^*Rosa26*^N1icd/+^ (KPN)	Jackstadt et al., (2019)	N/A
*Mouse: villin*Cre^ER^; *Kras*^G12D/+^; Tr*p53*^fl/fl^(KP)	Jackstadt et al., (2019)	N/A
*Mouse: villin*Cre^ER^; *Apc*^fl/fl^; *Kras*^G12D/^+;*Trp53*^fl/fl^*TgfbrI*^fl/fl^ (AKPT)	Jackstadt et al., (2019)	N/A
C57BL/6J-*Apc*^*Min*^/J	The Jackson Laboratory	Stock No: 002020
*villinCreER, Tg(Vil-cre/ERT2*)23Sy	el Marjou et al., 2004	N/A
Apc (floxed), Apctm1Tno	Shibata and Toyoma (1997)	N/A
*Rosa26-Notch1 ICD, Gt(ROSA)26Sortm1(Notch1)Dam*	Murtaugh et al. (2003)	N/A
p53 (floxed), Trp53tm1Brn	(Marino et al., 2000)	N/A
Kras (G12D); Krastm4Tyj	Jackson et al. (2001)	N/A
Tgfbr1 (floxed), tm1Kar	Larsson et al. (2001)	N/A
*Braf*^*LSL-V600E/+*^	Mercer et al. (2005)	N/A
Lgr5tm2(DTR/EGFP)Fjs	Tian et al. (2011)	N/A

**Software and Algorithms**		

ISCindex	This paper	https://doi.org/10.5281/zenodo.6473396
ImageJ	Schneider et al. (2012)	https://ImageJ.nih.gov/ij/
QuPath	Bankhead et al. (2017)	https://qupath.github.io
HALO Image analysis software	Indica labs	https://indicalab.com/halo/
BWA-MEM	Li (2013)	http://bio-bwa.sourceforge.net
BBTools ver. 38.46	Bushnell (2014)	sourceforge.net/projects/bbmap/
STAR ver. 2.7.0f	Dobin et al. (2013)	https://github.com/alexdobin/STAR/releases/tag/2.7.0f
featureCounts ver. 1.6.4	Liao et al. (2014)	http://subread.sourceforge.net
FastQC version 0.11.8		http://www.bioinformatics.babraham.ac.uk/projects/fastqc
Trim Galore version 0.6.4.		https://www.bioinformatics.babraham.ac.uk/projects/trim_galore/
HISAT2 version 2.1.0	Kim et al., 2019, Koppens et al., 2021	http://daehwankimlab.github.io/hisat2/
affy package v1.56.0	Gautier et al. (2004)	http://daehwankimlab.github.io/hisat2/
SingleR	Aran et al. (2019)	https://bioconductor.org/packages/release/bioc/html/SingleR.html
scran	Lun et al. (2016)	https://bioconductor.org/packages/release/bioc/html/scran.html
Seurat	Satija et al. (2015)	https://satijalab.org/seurat/
GSVA ver 1.38.2	Hänzelmann et al. (2013)	https://bioconductor.org/packages/release/bioc/html/GSVA.html
EdgeR ver. 3.32.1	Robinson (2010)	https://bioconductor.org/packages/release/bioc/html/edgeR.html
Limma ver 3.46.0	Ritchie et al. (2015)	https://bioconductor.org/packages/release/bioc/html/limma.html
*SIFT*	Ng and Henikoff (2003)	https://sift.bii.a-star.edu.sg
*PolyPhen*	Ramensky et al. (2002)	http://genetics.bwh.harvard.edu/pph/
*copynumber*	Nilsen et al. (2012)	https://bioconductor.org/packages/release/bioc/html/copynumber.html
TCGAbiolinks 2.18.0		https://bioconductor.org/packages/release/bioc/html/TCGAbiolinks.html
survival 3.2-7		https://cran.r-project.org/web/packages/survival/index.html

**Other**		

TCGA RNA-seq, mutation, and copy number alteration data from colon and rectal adenocarcinomas (TCGA-COADREAD)	GDC data portal	https://gdc.cancer.gov/
Human colon single cell RNA sequencing data	Lee et al. (2020)	GEO: GSE132257 GEO: GSE144735
Gene expression microarray data from colorectal tumors	Jorissen et al. (2009)	GEO: GSE14333
Gene expression microarray data from colon cancers	Marisa et al. (2013)	GEO: GSE39582
Single cell RNA-seq data from mouse small intestinal organoids grown in Matrigel or on collagen	Ramadan et al. (2021)	https://github.com/davidhuels/collagen_matrigel_project
RNA-seq data from mouse epithelial cells grown in Matrigel or Type I collagen	Yui et al. (2018)	ArrayExpress: E-MTAB-5247
RNA-seq data from mouse small intestinal organoids grown in Matrigel or on Type I collagen	Ramadan et al. (2021)	ArrayExpress: E-MTAB-10082
Gene expression microarray data from multiple random non-adjacent biopsies of colon tumors	Ubink et al. (2017)	GEO: GSE85043

### Resource availability

#### Lead contact

Further information and requests for resources and reagents should be directed to and will be fulfilled by the lead contact, Simon Leedham (simon.leedham@well.ox.ac.uk).

#### Materials availability

Mouse lines and organoids generated in this study are available with an MTA.

### Experimental model and subject details

#### Animals

The study comprised the use of two mouse cohorts: an internal mouse i.e. Oxford cohort and the ACRCelerate cohort. All procedures were carried out in accordance to Home Office UK regulations and the Animals (Scientific Procedures) Act 1986. All mice are housed in individually ventilated cages at the animal unit either at the Functional Genetics Facility (Wellcome Centre for Human Genetics, University of Oxford) or The Beatson Institute (Glasgow). All mice were housed in a specific-pathogen-free (SPF) facility, with unrestricted access to food and water, and were not involved in any previous procedures. All strains used in this study were maintained on C57BL/6J background for ≥6 generations. All procedures were carried out on mice of at least 6 weeks of age, both male and female.

#### Human subjects

Samples were collected from four cohorts: S:CORT cancer cohort, Oxford BRC cancer cohort, pre-cancer polyps cohort and colitis cohort. All human samples were obtained following ethical approval and individual informed consent (Ethics No 16/NI/0030 and 15/EE/0241 for S:CORT cohorts, REC: 17/NW/0252 BRC cancer cohort, and Oxford GI biobank ethics 18/SL/JE/Early-detection and OCHRe:18/A11 for the colitis cohort). All samples were subject to expert histopathological review. Data from the FOXTROT trial, developed by the NCRI Colorectal Cancer Clinical Studies Group (NCT00647530) has been used in this study.

#### Organoid models

To grow mouse organoids, tumor fragments were isolated from *villin*Cre^ER^; *Kras*^G12D/+^; Tr*p53*^fl/fl^*Rosa26*^N1icd/+^ (KPN organoids), *villin*Cre^ER^; *Kras*^G12D/+^; Tr*p53*^fl/fl^ (KP organoids), and *villin*Cre^ER^; *Apc*^fl/fl^; *Kras*^G12D/^+; *Trp53*^fl/fl^*TgfbrI*^fl/fl^ mice (AKPT organoids), while small intestine tissue fragments were isolated from wild-type C57BL/6J mice (WT organoids). Isolation was performed from both male and female mice at age 6–12 weeks. Details on mouse organoid derivation and maintenance can be found in the detailed methods.

### Methods Details

#### Treatment of animals

The mouse alleles used in this study are listed in the Key Resources Table. Phenotype induction was obtained by intraperitoneal tamoxifen (Merck) injection in the inducible models. *Lgr5*+ cells in the mice were ablated with a single intraperitoneal dose of DT (Cat # 322326-1mg, Merck) in saline (50 μg kg^−1^).

#### Formalin-fixed paraffin embedded processing

Gut preparations were washed in PBS, fixed overnight in 10% neutral buffered formalin and then transferred to 70% ethanol prior to processing for embedding. Formalin**-**fixed gut sections were rolled into Swiss Rolls, pinned and placed in a histology cassette. Specimens were processed using a Histomaster machine (Bavimed). Processed samples were embedded in paraffin wax using a paraffin embedding station (EG1150H, Leica).

#### Nucleic acid extraction

For the Oxford mouse cohort, RNA was extracted using the RNeasy Micro Kit from Qiagen (74004) and DNase treatment was performed using the DNA-free kit from Life Technologies (AM1906).

For the ACRCelerate mouse cohort, RNA extraction was performed using a Qiagen RNeasy kit according to the manufacturer’s protocols.

For formalin-fixed paraffin embedded (FFPE) samples from human cohorts, 2–10 5 micron sections were extracted using the High Pure FFPE RNA Isolation kit (Roche Life Sciences, Penzberg, Germany) under RNase free conditions following the manufacturer’s protocol. RNA quantity and quality were assessed using the RNA Qubit Assays (High sensitivity and Broad range, Thermofisher) and by Nanodrop, respectively.

#### RNA sequencing

Transcriptomic profiling was carried out by 3′RNAseq for the Oxford mouse cohort, pre-cancer polyps and the colitis cohorts. Libraries were sequenced on an Illumina HiSeq4000 instrument (Illumina). 3′RNAseq libraries were prepared using the QuantSeq 3′ mRNA-Seq Library Prep Kit FWD for Illumina (Lexogen, Austria). The manufacturer’s protocol for FFPE or fresh frozen tissue was used based on the source tissue material. For murine sample libraries, RNA input varying from 20 to 1000ng and 14–20 PCR cycles were used. For human sample libraries, RNA input varying from 53 to 10,000ng and 16–21 PCR cycles were used. Library quality and quantity were assessed using the HS 1000 DNA TapeStation (Agilent) and DNA High Sensitivity Qubit Assay (ThermoFisher) respectively, before pooling and sequencing on an Illumina HiSeq4000 instrument (Illumina). Raw sequence reads were subjected to adapter trimming using *BBduk* (*BBTools* ver. 38.46). Trimmed reads were aligned to the GRCh38 build of the human reference (for human data) or to the GRCm38 build of the mouse reference (for mouse data) using *STAR* (ver. 2.7.0f). Ensembl 96 annotations were used for alignment and subsequent quantifications. Gene expression was quantified using *featureCounts* (ver. 1.6.4).

For the ACRCelerate cohort, libraries were prepared using a TruSeq RNA sample prep kit v2 (Illumina) and sequenced on an Illumina NextSeq using the High Output 75 cycles kit (2x36 cycles, paired end reads, single index). Raw sequence quality was assessed using FastQC version 0.11.8, then sequences were trimmed to remove adaptor sequences and low-quality base calls, defined as those with a Phred score of less than 20, using Trim Galore version 0.6.4. Trimmed sequences were aligned to mouse genome build GRCm38.98 using HISAT2 version 2.1.0 and raw counts per gene were determined using *featureCounts* version 1.6.4. Counts were then normalized via quantile normalization in R.

#### Microarray transcriptome profiling

The S:CORT cancer cohort transcriptomes were analyzed by microarray. Extracted RNA was first amplified using the NuGen Ovation FFPE Amplification System v3 (NuGen San Carlos, California, USA). The amplified product was hybridized to the Almac Diagnostics XCEL array (Almac, Craigavon, UK), a cDNA microarray-based technology optimized for archival FFPE tissue, and analyzed using the Affymetrix Genechip 3000 7G scanner (Affymetrix, Santa Clara, California, USA) as previously described (12). Microarray data were quality checked then pre-processed where raw CEL files underwent the Robust Multiarray Average normalization for the Almac Diagnostic XCEL array with the affy package (v1.56.0) (17).

#### *In situ* hybridization

For *in situ* hybridization (ISH) of both human and mouse FFPE samples, 4 μm formalin-fixed, paraffin-embedded tissue sections were used. The sections were baked at 60°C for 1 h before dewaxing in xylene and ethanol. Fluorescent *ISH* was then performed using the RNAscope Fluorescent Multiplex Reagent Kit (Bio-techne) in accordance with the supplier’s guidelines. Probes were purchased from Bio-techne: Mm-Anxa1 (509291), Hs-ANXA1 (465411), Mm-Ly6a-C2 (427571-C2), Hs-PLAUR (542701), Mm-Clu-C3 (427891-C3), Hs-CLU (606241), Mm-Lgr5-C2 (312171-C2), Hs-LGR5-C2 (311021-C2).

#### Immunohistochemistry

Sections were de-paraffinized in xylene and rehydrated through graded alcohols to water. Antigen retrieval was done by pressure cooking in 10 mmol/L citrate buffer (pH 6.0) for 5 min. Endogenous peroxidase activity was blocked by incubating in 3% hydrogen peroxidase (in methanol) for 20 min. Next, sections were blocked with 1.5% serum for 30 min, after which they were incubated with primary antibodies for 2 h. Antibodies against the following proteins were used: KI67 (Cell Signaling Technology, CS12202S, 1/500), Caspase 3 (R&D Systems AF835, 1:800). The sections were then incubated with appropriate secondary antibodies for 30 min at room temperature. For chromogenic visualization, sections were incubated with ABC (Vector labs) for 30 min and stained using DAB solution (VectorLabs), after which they were counterstained with hematoxylin, dehydrated and mounted.

#### Multiplex immunofluorescence

Multiplex immunofluorescence (MPIF) staining was performed on FFPE sections of thickness 4-μm using the OPAL protocol (Akoya Biosciences, Marlborough, MA) on the Leica BOND RXm autostainer (Leica Microsystems, Wetzlar, Germany). Six consecutive staining cycles were performed using the following primary antibody-Opal fluorophore pairs:

**Immune panel:** (1) Ly6G (1:300, 551459; BD Pharmingen)–Opal 540; (2) CD4 (1:500, ab183685; Abcam)–Opal 520; (3) CD8 (1:800, 98941; Cell Signaling)–Opal 570; (4) CD68 (1:1200, ab125212; Abcam)–Opal 620; (5) FoxP3 (1:400, 126553; Cell Signaling)–Opal 650; and (6) E-cadherin (1:500, 3195; Cell Signaling)–Opal 690.

**Stroma panel:** (1) Gremlin 1 (1:750, AF956; R&D)–Opal 540; (2) CD34 (1:3000, ab81289; Abcam)–Opal 520; (3) CD146 (1:500, ab75769; Abcam)–Opal 570; (4) SMA (1:1000, ab5694; Abcam)–Opal 620; (5) Periostin (1:1000, ab227049; Abcam)–Opal 690; and (6) E-cadherin (1:500, 3195; Cell Signaling)–Opal 650.

**Matrix panel:** (1) Laminin (1:400, ab11575; Abcam)-Opal 540; (2) Tenascin-C(1:600, ab108930; Abcam)-Opal 520; (3) Fibronectin (1:1000, F3648; Sigma-Aldrich)-Opal 570; (4) Osteopontin (1:750, ab218237; Abcam)-Opal 620; MMP3 (1:100,ab52915; Abcam)-Opal 650; (5) Collagen I (1:400, 72026; Cell Signaling)-Opal 690.

Tissues sections were incubated for 1 h in primary antibodies and detected using the BOND Polymer Refine Detection System (DS9800; Leica Biosystems, Buffalo Grove, IL) in accordance with the manufacturer’s instructions, substituting DAB for the Opal fluorophores, with a 10-min incubation time and withholding the hematoxylin step. Antigen retrieval at 100°C for 20 min, in accordance with standard Leica protocol, with Epitope Retrieval Solution one or two was performed prior to each primary antibody being applied. Sections were then incubated for 10 min with spectral DAPI (FP1490, Akoya Biosciences) and the slides mounted with VECTASHIELD Vibrance Antifade Mounting Medium (H-1700-10; Vector Laboratories). Whole-slide scans and multispectral images (MSI) were obtained on the Akoya Biosciences Vectra Polaris. Batch analysis of the MSIs from each case was performed with the inForm 2.4.8 software provided. Finally, batched analyzed MSIs were fused in HALO (Indica Labs) to produce a spectrally unmixed reconstructed whole-tissue image. Cell density analysis was subsequently performed for each cell phenotype across the three MPIF panels using HALO.

#### Single-cell RNAseq analysis

Previously published human colon single cell RNA sequencing data was used (Lee et al., 2020). The raw expression matrix from the *CellRanger* pipeline was normalized with *Seurat*. Cell type assignment was performed with *SingleR* (Aran et al., 2019). Epithelial cells were retained for downstream analysis and quality control filtering was applied for >1000 unique molecular identifier counts (UMI), <30% mitochondrial gene expression, minimum 2,000, and maximum 6,000 detected genes. Dimension reduction was performed with UMAP of highly variable genes, defined by variance modeling (function *modelGeneVar()*) at an FDR<5%, after excluding mitochondrial and ribosomal genes. We applied the mutual nearest-neighbor algorithm for dataset integration (Haghverdi et al., 2018).

Mouse genes were mapped to the most confident human orthologs, based on the Ensembl annotation (March 2020 version). The genes were further classified into epithelial, non-epithelial and non-specific, according to the specificity of their expression in the KUL3 colorectal single-cell dataset (Lee et al., 2020). Briefly, we aggregated raw counts from cells of similar type (epithelial, stroma, myeloid, lymphocytes, endothelial and other) into pseudobulk with the *muscat* package. If the expression of a gene was higher in a given cell type than in all other cell types by at least one raw count and that difference was always statistically significant at p < 0.01, that gene was considered specific for that cell type. Thus, only genes of predominantly epithelial origin were kept from every signature. The mean expression value was used as the signature value for every cell.

The proportion of true LGR5 positive CBC cells was expected to be close to 5%, according to previous studies (Cao et al., 2020). Therefore, the cutoff for CBC positivity was fixed at the 95th percentile of the signature values in the cell population (rounded to 0.45 logcounts). There was no *a priori* expectation for the proportion of true RSC positive cells, so on the assumption that the number of RSC positive stem cells would be comparable, the cutoff for RSC positivity was also fixed at the 95th percentile (rounded to 0.65 logcounts). Double-positive (“mixed”) cells were defined as having both RSC and CBC expression above the predefined cutoffs.

#### Tumor organoid generation and maintenance

Organoid growth media was made from advanced DMEM/F12 supplemented with penicillin (100 U/ml) and streptomycin (100 μg/mL) (ThermoFisher Scientific, 15140122), 2 mM L-glutamine (ThermoFisher Scientific, 25030081) 10 mM HEPES (ThermoFisher Scientific, 15630080), N2-supplement (ThermoFisher Scientific, 17502001), B27-supplement (ThermoFisher Scientific, 17504044), recombinant human EGF 50 ng/mL (Peprotech, AF-100-15) and recombinant murine Noggin 100 ng/mL (Peprotech, 250-38).

Tumor fragments from mice were taken in ice-cold PBS at time of dissection. Fragments were then cut into 2–5mm pieces and washed in ice-cold PBS three times. These were then incubated in 5mL of 10x Trypsin and 200U recombinant DNase at 37°C for 30 min after being shaken vigorously. Next, 20mL of ADF was added and fragments were shaken vigorously again. Samples were spun and supernatant was aspirated. The pellet was resuspended in 10mL of ADF and passed through a 70um strainer. Tube and strainer was then rinsed with another 5mL of ADF. The filtered suspension was pelleted and supernatant aspirated. This was suspended in Matrigel and plated depending on pellet size. The plate was inverted and left to set at 37°C for 10 min before adding standard growth media. Organoids were grown at 37°C 5% CO_2_ 21% O_2_.

Organoids were passaged every 2–3 days using mechanical dissociation and split 1:3 or 1:2 ratio depending on organoid density. Organoid lines were frozen in Gibco cell culture freezing media and revived before use for experiments. Routine mycoplasma testing was done before transplantation experiments. For RNA seq, organoids were washed in PBS, pelleted and snap frozen on dry ice.

#### Organoid treatment with Interferon-gamma and TGFβ

Wild-type mouse colonic organoids were plated onto 24-well plates in Matrigel and allowed to culture for 5 days before treatment with recombinant murine IFN-γ (PeproTech, UK) at concentrations of 0.2, 1, and 5 ng/mL 24 h after treatment RNA was extracted and cDNA synthesized, and the target genes Lgr5 and Ly6a, GAPDH were measured by qRT-PCR.

Mouse intestinal organoids (wild-type, AKPT, and KPN) were plated onto 24-well plates in Matrigel and cultured for 3 days before treatment with either recombinant murine IFN-γ (PeproTech, UK) at 1 ng/mL or recombinant murine TGFβ 1 (R&D, UK) at 5 ng/mL. RNA was extracted and processed for RNA sequencing 24 h after treatment.

#### FACS sorting of IFN-γ treated Lgr5-GFP organoids

For FACS analysis of IFN-γ-treated organoids, adult *Lgr5;EGFP;CreERT2* organoids were grown in matrigel as previously described (Sato et al. 2009). Interferon gamma (R&D rmIFN-gamma #485-MI, 5 ng/ml) was added on day 5 after passaging. After 24 h, organoids were dissociated into single cells by incubation with 1mL TrypLE Express (Gibco) for 15 min at 37C with mechanical dissociation after 10 min. Cells were then incubated with anti-mouse Ly6a-APC (1/1000, eBioscience #17-5981-81) for 30 min on ice. Hoechst 33342 was added to exclude live from dead cells and an Iso-type control (APC) was used for gating.

#### Stem cell sorting and single-cell clonogenicity analysis

Stem cell populations were sorted from KPN mice. Mice were sacrificed upon development of intestinal phenotype. The intestinal tissue was removed, and subjected to manual dissociation followed by enzymatic dissociation with 5mL 10x Trypsin (5 mg/ml, Gibco), 1x DNase buffer (500ul) and 200U recombinant DNase I (20ul, Roche, 04716728001) at 37°C in a shaker set at 100 RPM. The cell suspension was then incubated in Mouse Fc Block purified anti-mouse CD16/CD32 mAb, then stained for the following markers: CD31, CD45, EpCAM, Ly6a and Ephb2. The stained cells were sorted for the following populations on a BD FACSAria Fusion Flow Cytometer: (1) Ly6a+Ephb2+, (2) Ly6a+Ephb2-, (3) Ephb2+Ly6a−, and (4) Ly6a-Ephb2−. The gating strategy used is shown in Figure S2. The cells were collected in media on 1mL Eppendorf tubes before being grown in matrigel supplemented with Jagged1 (Anaspec, 1 μM) and cultured in advanced DMEM/F12 (Thermo Fisher Scientific) supplemented with GlutaMAX (Thermo Fisher Scientific, 1%) and Penicillin/Streptomycin (Thermo Fisher Scientific, 0.5%) in the presence of human EGF (Peprotech; 50 ng/mL), murine Noggin (Peprotech; 100 ng/mL), human R-spondin1 (R&D; 500 ng/mL), murine Wnt3a (Cell guidance systems; 100 ng/ml), Chir99021 (Stemgent; 3 microM), Prostaglandin E2 (PGE2; Sigma; 2.5microM), Nicotinamide (Sigma; 10 mM), N-2 supplement (Thermo Fisher Scientific; 1%) and B-27 supplement (Thermo Fisher Scientific; 2%). In the first 3 days, cells were provided with Y-27632 as additional supplement. Cells were imaged at day 7 from plating and number of organoids manually counted.

### Quantification and statistical analysis

#### Calculation of the stem cell index

The RSC gene signature was derived from the repair signature in Yui et al. (2018) and the spheroid upregulated gene list from Mustata et al. (2013). Cell-type-associated expression was characterized using hierarchical clustering of signature genes in published single cell RNAseq data from colorectal tumors and normal colon tissue (Lee et al., 2020), and genes from clusters that represented epithelial-expressed genes with high stromal expression, stromal-expressed genes with no distinctive expression in other cell types, as well as genes with no clear cell-type-specific expression were excluded from the signature. The resulting gene signature of 265 genes is provided in Table S1.

The CBC signature was taken from the *Lgr5* intestinal stem cell signature in Munoz et al. (2012).

Single sample enrichment scores for the RSC and CBC signatures were calculated using Gene Set Variation Analysis (GSVA (Hänzelmann et al., 2013) from TMM-normalized CPM values in RNAseq data and from mean per gene values in microarray data. The stem cell index was calculated by subtracting the CBC score from the RSC score.

#### Quantification of Casp3 and Ki67 positive cells

Casp3 and Ki67 positive cells were quantified using QuPath digital pathology software (v0.2.3, (Bankhead et al., 2017)), downloaded from https://QuPath.github.io/. Firstly, annotations of polyp areas were created for each tissue sample with areas of folded tissue excluded to eliminate false positive signals. Cells were identified within QuPath using a custom algorithm established via stain separation using color reconstruction. Positive cell detection analysis was run to identify DAB positive cells for both Casp3 and Ki67 stained sections and results reported as percentage of positive cells. Each annotation was manually verified for correct signal identification.

#### Gene set enrichment analysis

Gene Set Enrichment Analysis was performed using the *fgsea* package. Single sample enrichment scores were calculated using Gene Set Variation Analysis (GSVA, (Hänzelmann et al., 2013).

The signatures used were previously published: Yap (Gregorieff et al., 2015; Yui et al., 2018), Wnt (Sansom et al., 2004; Van der Flier et al., 2007), Kras (Loboda et al., 2010), IFN-γ (Ayers et al., 2017), Fibroblast TGF-β response (Calon et al., 2012), and the MSigDB Hallmark gene set (Liberzon et al., 2015) and Reactome (Jassal et al., 2020). Mouse genes were mapped to the most confident human orthologs, based on the Ensembl annotation (March 2020 version).

#### Mutational and copy number alteration analysis on TCGA colorectal cohort

Mutation Annotation Format files generated by SomaticSniper and mean segment copy number calls from the TCGA Colorectal adenocarcinoma project (TCGA-COADREAD) were downloaded from the GDC data portal. Mutations were filtered for pathogenicity using the *SIFT* and *PolyPhen* predictors and subsequent analysis were conducted using R and results were plotted using the R function *barplot*. For copy number analysis, CNV values smaller than −0.3 were categorized as a loss (−1), whereas values above 0.3 were annotated as gains (+1). Gains and losses were plotted using the *plotFreq* function from the R package *copynumber*.

#### Survival analyses

TCGA-COADREAD RNA sequencing and clinical data were downloaded from the Genomics Data Commons using TCGAbiolinks on R. Gene expression microarray and clinical data from GSE14333 (Jorissen et al., 2009) GSE39582 (Marisa et al., 2013) were downloaded from GEO. Patients with Stage 1-3/Dukes A-C cancers and with no missing data in any covariate were included in the analysis - 440 in the TCGA-COADREAD data, 226 in the Jorissen et al. data, and 513 in the Marisa et al. data. The relationship between quintiles of stem cell index and survival (Progression-free survival/PFS in the TCGA-COADREAD data and Disease-free survival/DFS in the Jorissen et al. and Marisa et al. data) was examined using multivariate Cox regression analyses with TNM/Dukes stage, age, and gender as possible confounding factors. Survival analyses were performed in R using the survival package.

#### Analysis of therapy response

Associations of stem cell phenotype to therapy response were investigated in data from the FOXTROT trial. The analysis was undertaken on 75 patients from Trial Arm A, who were given 6 weeks of pre-operative OxFP chemotherapy followed by surgery then 18 (or 6) weeks post-operative OxFP chemotherapy. For each of the patients, pre-treatment biopsies and a post-treatment resection sample were collected for genetic and transcriptomic profiling.

Response to treatment of patients was determined by histological assessment. Patients with no regression (no change) or mild regression (residual cancer cells are higher than fibrosis) were classified as non-responders, while those with marked regression (presence of fibrosis with rare residual cancer cells) or moderate regression (increased presence of residual cancer cells but still high fibrosis) were classified as responders.

The stem cell index was for each sample. Samples with a positive Index score were classified as RSC-positive. Samples with a negative Index score were classified as CBC-positive. Patients were discerned into: those with a static stem cell phenotype (where pre- and post-treatment samples were either both RSC-positive or both CBC-positive) and those with a plastic stem cell phenotype (where the tumor shifted either from being RSC-positive pre-treatment to CBC-positive post-treatment, or from CBC-positive pre-treatment to RSC-positive post-treatment). Differences in treatment response between static and plastic stem cell phenotypes were evaluated using a Fisher exact test.

## Data Availability

• The RNA-seq data generated for this paper have been deposited at the ArrayExpress database at EMBL-EBI (www.ebi.ac.uk/arrayexpress) and are publicly available as of the date of publication. Accession numbers are listed in the key resources table. Publicly available data were analyzed in this paper. The repositories containing the datasets and their respective accession numbers are listed under the “Other” section in the key resources table. • A function to calculate the stem cell index has been made available in an R package *ISCindex* (https://github.com/gnvalbuena/ISCindex). All original code has been deposited at Zenodo and is publicly available as of the date of publication. The DOI is listed in the key resources table. • Any additional information required to reanalyze the data reported in this paper is available from the lead contact upon request.

## Supplemental information

Document S1. Figures S1–S5

Table S1. RSC gene list, related to STAR Methods

Table S2. Reactome pathways upregulated or downregulated between top and bottom stem cell index tertiles of mouse models, related to Figure S3D

Document S2. Article plus supplemental information
