# Dynamic and adaptive cancer stem cell population admixture in colorectal neoplasia

**DOI:** 10.1016/j.stem.2022.09.005

**Published:** 2022-11-03

**Authors:** Ester Gil Vazquez, Nadia Nasreddin, Gabriel N. Valbuena, Eoghan J. Mulholland, Hayley L. Belnoue-Davis, Holly R. Eggington, Ryan O. Schenck, Valérie M. Wouters, Pratyaksha Wirapati, Kathryn Gilroy, Tamsin R.M. Lannagan, Dustin J. Flanagan, Arafath K. Najumudeen, Sulochana Omwenga, Amy M.B. McCorry, Alistair Easton, Viktor H. Koelzer, James E. East, Dion Morton, Livio Trusolino, Timothy Maughan, Andrew D. Campbell, Maurice B. Loughrey, Philip D. Dunne, Petros Tsantoulis, David J. Huels, Sabine Tejpar, Owen J. Sansom, Simon J. Leedham

## Main text

(Cell Stem Cell *29*, 1213–1228, August 4, 2022)

In the version of our manuscript that was accepted by the Cell Press editorial office, we mistakenly included a misspelling of the first author’s surname. We did not catch this unfortunate error during the production process, and the manuscript published with the error included. We have now corrected the spelling, and the correct author list appears here and in the online version of our article. We apologize for the oversight.

