# Supplementary material for: Dynamic and adaptive cancer stem cell population admixture in colorectal neoplasia
Source: Cell Stem Cell. 2022 Aug 4;29(8):1213–1228.e8. doi: 10.1016/j.stem.2022.07.008 (PMC9592560; doi:10.1016/j.stem.2022.07.008)
Supplement: Document S1. Figures S1–S5 [file mmc1.pdf]

Cell Stem Cell, Volume *29*

Supplemental Information

Dynamic and adaptive cancer stem cell population admixture in colorectal neoplasia

Ester Gil Vazquez, Nadia Nasreddin, Gabriel N. Valbuena, Eoghan J. Mulholland, Hayley L. Belnoue-Davis, Holly R. Eggington, Ryan O. Schenck, Valérie M. Wouters, Pratyaksha Wirapati, Kathryn Gilroy, Tamsin R.M. Lannagan, Dustin J. Flanagan, Arafath K. Najumudeen, Sulochana Omwenga, Amy M.B. McCorry, Alistair Easton, Viktor H. Koelzer, James E. East, Dion Morton, Livio Trusolino, Timothy Maughan, Andrew D. Campbell, Maurice B. Loughrey, Philip D. Dunne, Petros Tsantoulis, David J. Huels, Sabine Tejpar, Owen J. Sansom, and Simon J. Leedham

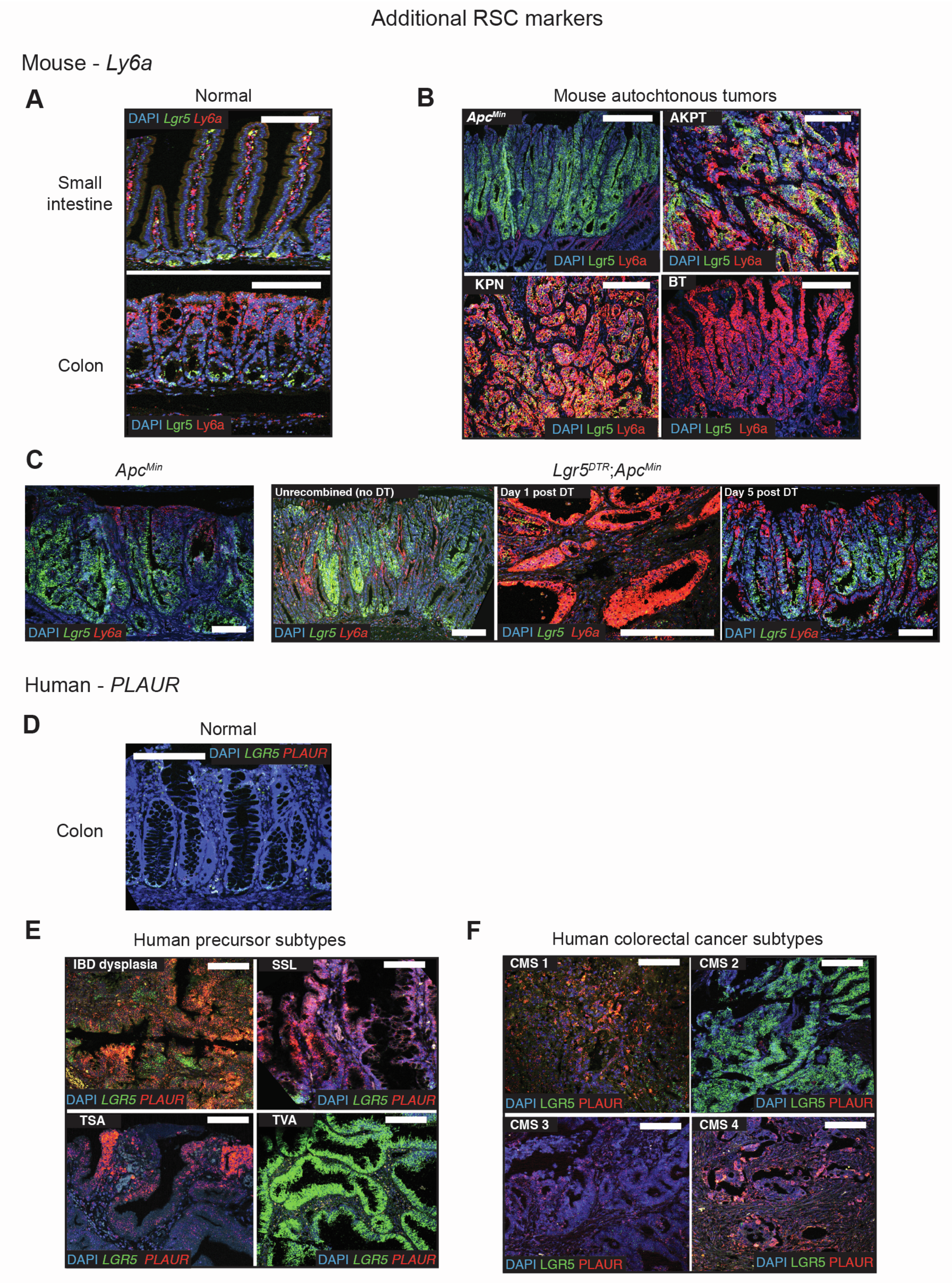

Figure S1. Additional RSC morphological markers, related to Figure 1. A. Dual color ISH for *Lgr5* (CBC marker - green) and *Ly6a* (RSC marker - red) expression in normal mouse small intestine and colon. **B.** Dual color ISH for *Lgr5* (CBC marker - green) and *Ly6a* (RSC marker - red) expression in representative genotype tumors across the CBC to RSC spectrum. **C.** Dual color ISH for *Lgr5* (CBC marker - green) and *Ly6a* (RSC marker - red) to show marker expression change before and after CBC cell ablation in *Lgr5*^{DTR};*Apc*^{Min} mice. Driver alleles initialisation - A is *Apc*$^{fl/+}$, *Apc*^{Min} is *Apc*^{Min}, B is *Braf*^{V600E}, K is *Kras*^{G12D}, P is *p53*$^{fl/fl}$, T is *Tgfβr1*$^{fl/fl}$, N is *Rosa26*$^{N1icd/+}$ **D.** Dual color ISH for *LGR5* (CBC marker - green) and *PLAUR* (RSC marker - red) expression in normal human colon. **E-F.** Dual color ISH for *LGR5* (CBC marker - green) and *PLAUR* (RSC marker - red) expression in **E.** representative human precursor lesions and **F.** representative human colorectal cancers segregated by consensus molecular subtype. Scale bars, 100 µm.

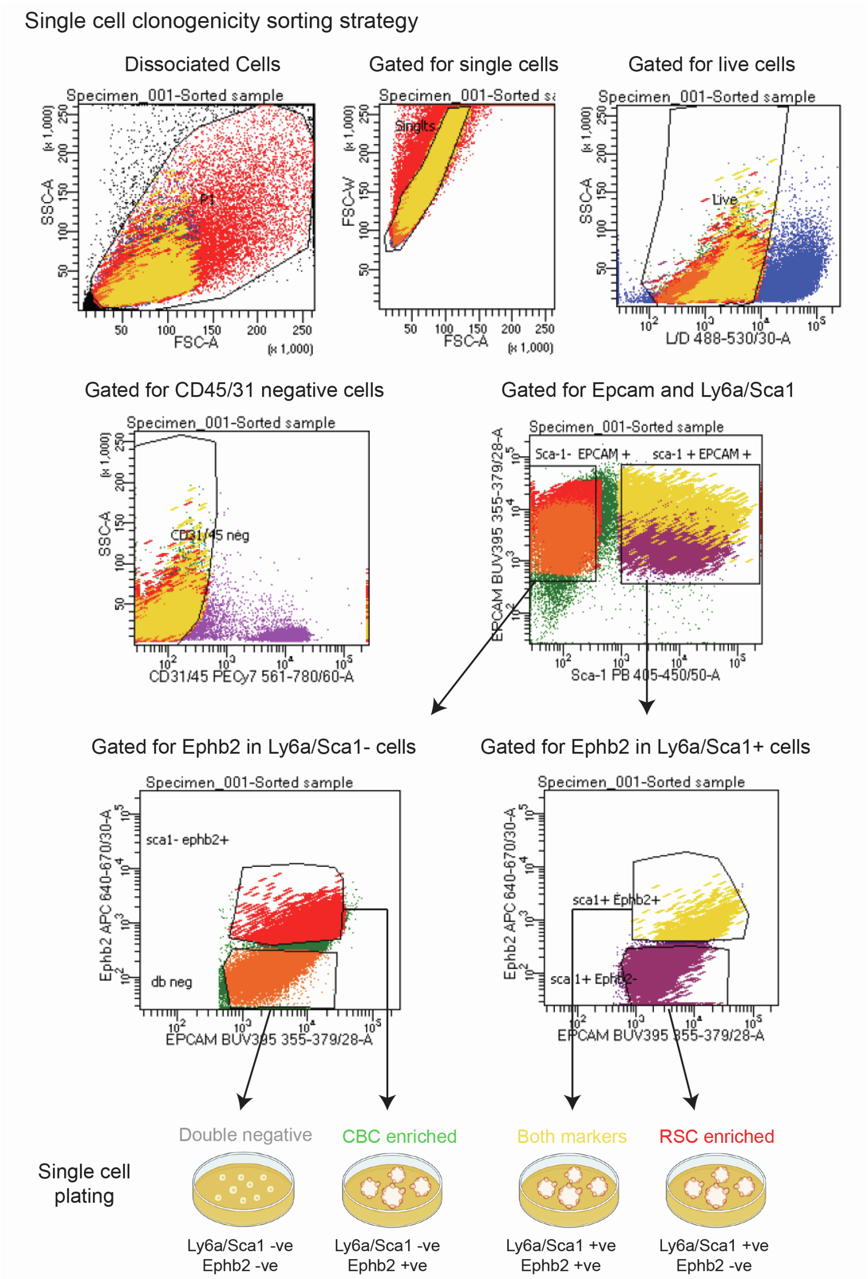

Figure S2. Single cell clonogenicity sorting strategy, related to Figure 1D. KPN mouse primary tumours (n=5) were disaggregated and cells sorted to generate live epithelial cells in four distinct groups, CBC-enriched, RSC-enriched, expression of both CBC and RSC markers, and double negative cells. Sorted single cells were plated in Matrigel and organoid cloning efficiency was assessed 7 days later.

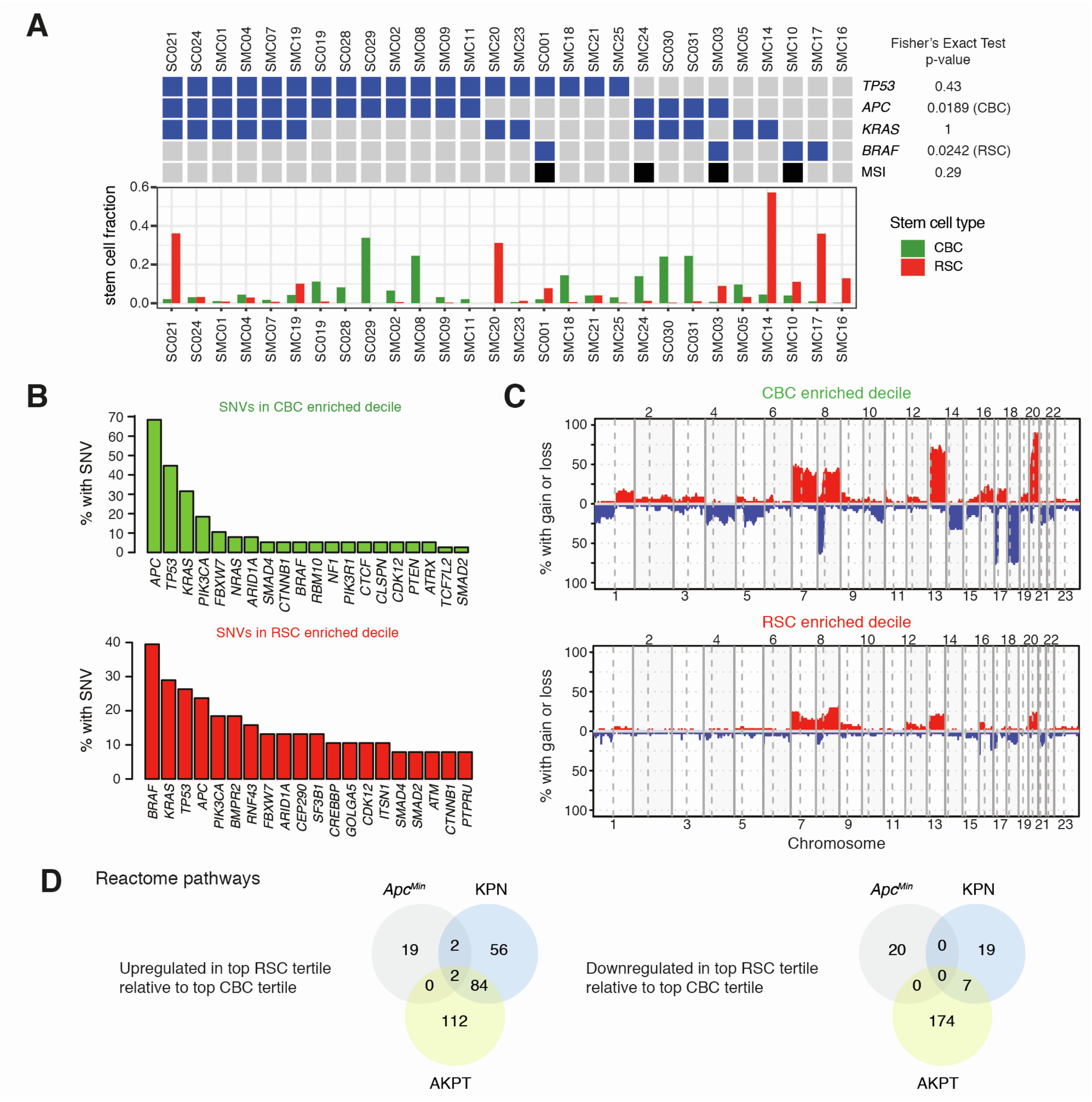

Figure S3. Human driver mutation landscape in CBC vs RSC enriched deciles, related to Figure 3. A. Fraction of epithelial cells with CBC or RSC phenotypes in single cell RNA sequencing data from human colorectal cancer patients. Mutation status of selected driver genes are marked in blue and MSI status is marked in black above each patient. Associations between CBC/RSC counts and mutation or MSI status were evaluated with a Fisher's Exact Test (p-values listed in figure). **B.** Comparison of most prevalent single nucleotide variant mutations in TCGA tumours subdivided into CBC and RSC predominant deciles **C.** Comparison of copy number variation by chromosome number in TCGA tumours subdivided into CBC and RSC predominant deciles. **D.** Comparison of similarities and differences between upregulated or downregulated Reactome pathways in *Apc*^{Min}, AKPT, or KPN mice from Gene Set Enrichment Analyses comparing the top tertile (highest stem cell index, most RSC) and the bottom tertile (lowest stem cell index, most CBC) of autochthonous tumors from each mouse genotype.

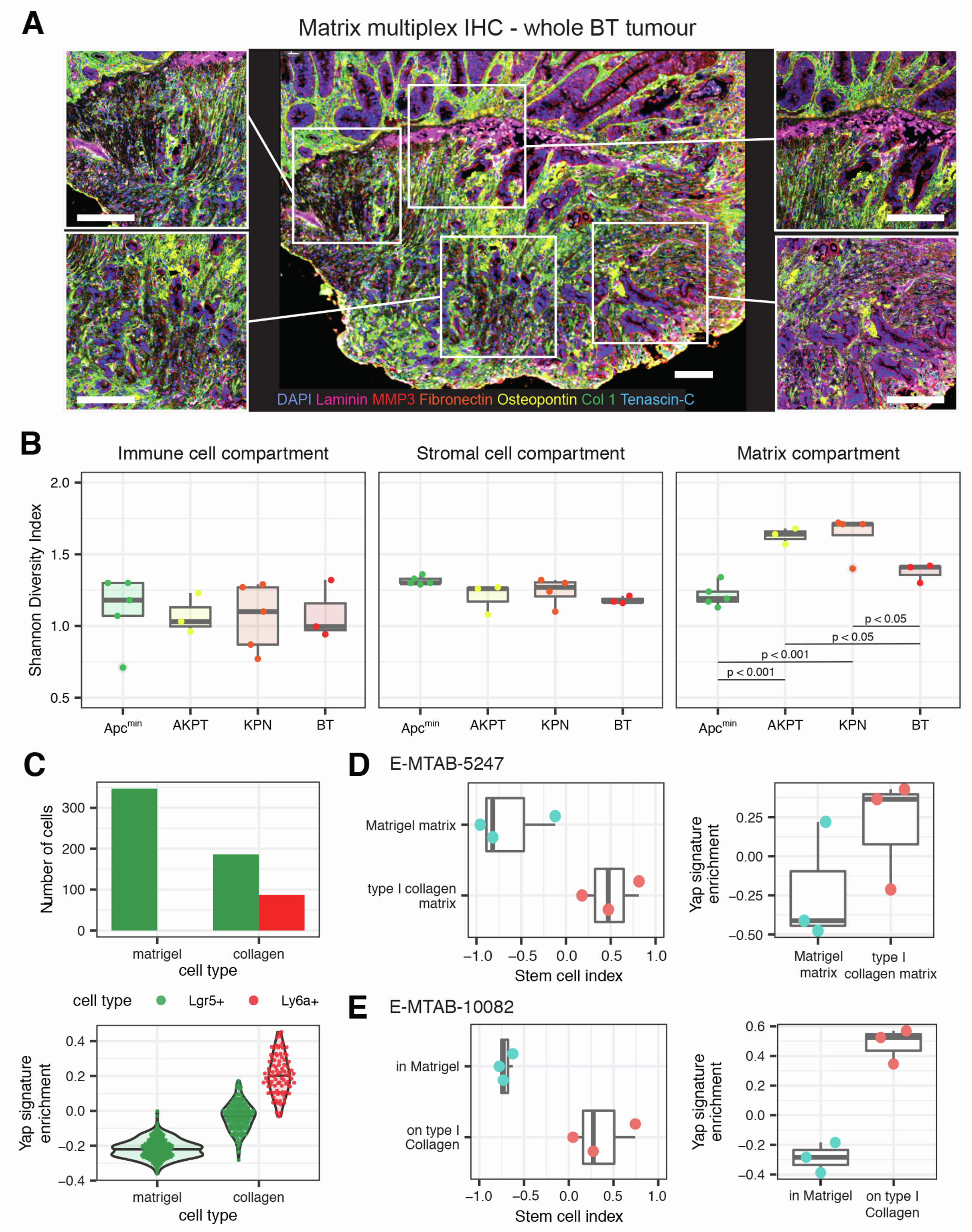

Figure S4. Matrix heterogeneity, diversity and impact of mechanotransduction on organoids, related to Figure 4. **A.** Topographical heterogeneity of matrix deposition detected using matrix multiplex IHC, across a representative whole tumour (BT mouse). Scale bars, 100µm **B.** Cross genotype diversity of immune, stromal cell populations and matrix assessed from quantification of multiplex IHC using Shannon's diversity index. Statistical analysis, ANOVA with Tukey post hoc test, p-values as stated. **C.** Number of *Lgr5*+ and *Ly6a*+ cells and Yap signature enrichment of single cells from organoids grown in either matrigel or collagen and analysed by single cell RNA-sequencing. **D-E.** Stem cell index and Yap signature enrichment of organoids grown in matrigel or in/on collagen analysed by bulk RNA-sequencing from **D.** E-MTAB-5247 (Yui *et al.*, 2018) and **E.** E-MTAB-10082 (Ramadan *et al.*, 2021).

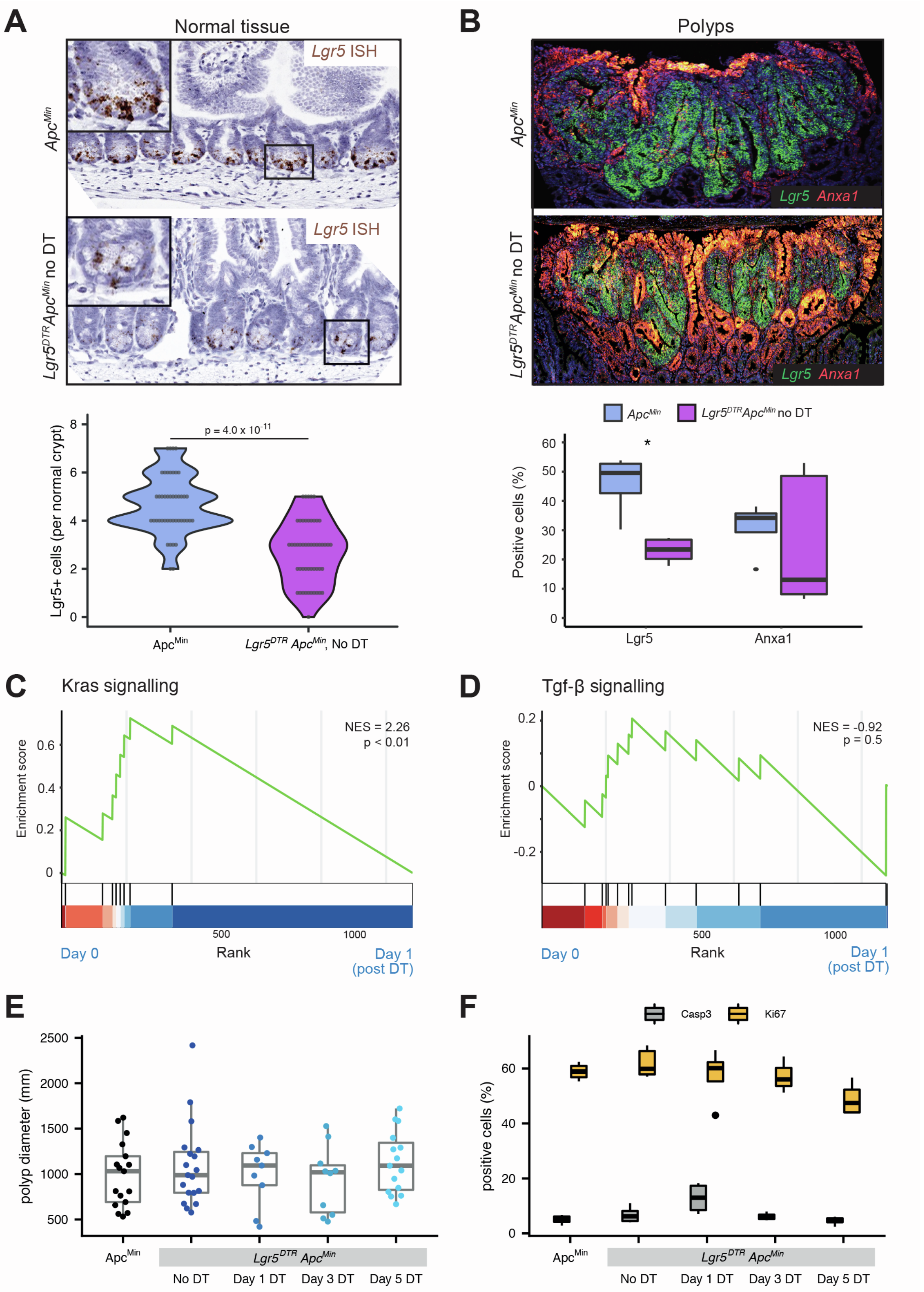

Figure S5. Impact of *Lgr5* hemizygosity and stem cell ablation on polyp size and proliferation in *$Lgr5^{DTR};Apc^{Min}$* mice, related to Figure 5. **A.** Chromogenic ISH and quantification showing impact of *Lgr5* hemizygosity on CBC cell count in unrecombined *$Lgr5^{DTR};Apc^{Min}$* mice. T-test, p value as stated. **B.** Dual colour fluorescent ISH and quantification showing decreased *Lgr5* expression in polyps in unrecombined *$Lgr5^{DTR};Apc^{Min}$* mice. **C-D.** Gene set enrichment analysis showing enrichment of **C.** Kras signalling and **D.** lack of enrichment of Tgfβ signalling between Day 0 (unrecombined) and Day 1 (after stem cell ablation) **E.** Polyp diameter (micrometres) and **F.** cell proliferation (assessed by quantified Ki67 stain) and apoptosis (assessed by quantified caspase staining) of polyps in *$Lgr5^{DTR};Apc^{Min}$* mice before and after CBC cell ablation.
